# Genetic Associations Between Modifiable Risk Factors and Alzheimer Disease

**DOI:** 10.1001/jamanetworkopen.2023.13734

**Published:** 2023-05-17

**Authors:** European Alzheimer’s & Dementia Biobank Mendelian Randomization (EADB-MR) Collaboration, Jiao Luo, Jesper Qvist Thomassen, Céline Bellenguez, Benjamin Grenier-Boley, Itziar de Rojas, Atahualpa Castillo, Kayenat Parveen, Fahri Küçükali, Aude Nicolas, Oliver Peters, Anja Schneider, Martin Dichgans, Dan Rujescu, Norbert Scherbaum, Deckert Jürgen, Steffi Riedel-Heller, Lucrezia Hausner, Laura Molina Porcel, Emrah Düzel, Timo Grimmer, Jens Wiltfang, Stefanie Heilmann-Heimbach, Susanne Moebus, Thomas Tegos, Nikolaos Scarmeas, Jordi Clarimon, Fermin Moreno, Jordi Pérez-Tur, María J. Bullido, Pau Pastor, Raquel Sánchez-Valle, Victoria Álvarez, Mercè Boada, Pablo García-González, Raquel Puerta, Pablo Mir, Luis M. Real, Gerard Piñol-Ripoll, Jose María García-Alberca, Jose Luís Royo, Eloy Rodriguez-Rodriguez, Hilkka Soininen, Teemu Kuulasmaa, Alexandre de Mendonça, Shima Mehrabian, Jakub Hort, Martin Vyhnalek, Sven van der Lee, Caroline Graff, Goran Papenberg, Vilmantas Giedraitis, Anne Boland, Delphine Bacq-Daian, Jean-François Deleuze, Gael Nicolas, Carole Dufouil, Florence Pasquier, Olivier Hanon, Stéphanie Debette, Edna Grünblatt, Julius Popp, Luisa Benussi, Daniela Galimberti, Beatrice Arosio, Patrizia Mecocci, Vincenzo Solfrizzi, Lucilla Parnetti, Alessio Squassina, Lucio Tremolizzo, Barbara Borroni, Benedetta Nacmias, Sandro Sorbi, Paolo Caffarra, Davide Seripa, Innocenzo Rainero, Antonio Daniele, Carlo Masullo, Gianfranco Spalletta, Julie Williams, Philippe Amouyel, Frank Jessen, Patrick Kehoe, Tsolaki Magda, Giacomina Rossi, Pascual Sánchez-Juan, Kristel Sleegers, Martin Ingelsson, Ole A. Andreassen, Mikko Hiltunen, Cornelia Van Duijn, Rebecca Sims, Wiesje van der Flier, Agustín Ruiz, Alfredo Ramirez, Jean-Charles Lambert, Ruth Frikke-Schmidt

**Affiliations:** 1 Department of Clinical Biochemistry, Copenhagen University Hospital–Rigshospitalet, Copenhagen, Denmark; 2 Univ. Lille, Inserm, CHU Lille, Institut Pasteur Lille, U1167–RID-AGE–Facteurs de risque et déterminants moléculaires des maladies liées au vieillissement, Lille, France; 3 Research Center and Memory clinic Fundació ACE, Institut Català de Neurociències Aplicades, Universitat Internacional de Catalunya, Barcelona, Spain; 4 Network Center for Biomedical Research in Neurodegenerative Diseases, National Institute of Health Carlos III, Madrid, Spain; 5 Division of Psychological Medicine and Clinical Neuroscience, School of Medicine, Cardiff University, Wales, United Kingdom; 6 Division of Neurogenetics and Molecular Psychiatry, Department of Psychiatry and Psychotherapy, Faculty of Medicine and University Hospital Cologne, University of Cologne, Cologne, Germany; 7 Department of Neurodegenerative diseases and Geriatric Psychiatry, University Hospital Bonn, Medical Faculty, Bonn, Germany; 8 Complex Genetics of Alzheimer's Disease Group, VIB Center for Molecular Neurology, Antwerp, Belgium; 9 Department of Biomedical Sciences, University of Antwerp, Antwerp, Belgium; 10 German Center for Neurodegenerative Diseases, Berlin, Germany; 11 Institute of Psychiatry and Psychotherapy, Charité–Universitätsmedizin Berlin, corporate member of Freie Universität Berlin, Humboldt–Universität zu Berlin, and Berlin Institute of Health, Institute of Psychiatry and Psychotherapy, Berlin, Germany; 12 German Center for Neurodegenerative Diseases, Bonn, Germany; 13 Department for Neurodegenerative Diseases and Geriatric Psychiatry, University Hospital Bonn, Bonn, Germany; 14 Institute for Stroke and Dementia Research, University Hospital, LMU Munich, Munich, Germany; 15 German Center for Neurodegenerative Diseases, Munich, Germany; 16 Munich Cluster for Systems Neurology, Munich, Germany; 17 Department of Psychiatry and Psychotherapy, Medical University of Vienna, Vienna, Austria; 18 Comprehensive Centre for Clinical Neurosciences and Mental Health, Medical University of Vienna, Vienna, Austria; 19 LVR-Hospital Essen, Department of Psychiatry and Psychotherapy, Medical Faculty, University of Duisburg-Essen, Essen, Germany; 20 Department of Psychiatry, Psychosomatics and Psychotherapy, Center of Mental Health, University Hospital of Würzburg, Würzburg, Germany; 21 Institute of Social Medicine, Occupational Health and Public Health (ISAP), University of Leipzig, Leipzig, Germany; 22 Department of Geriatric Psychiatry, Central Institute for Mental Health Mannheim, Faculty Mannheim, University of Heidelberg, Heidelberg, Germany; 23 Neurological Tissue Bank, Biobanc Hospital Clinic, Instituto de Investigaciones Biomédicas August Pi i Sunyer, Barcelona, Spain; 24 Alzheimer’s Disease and Other Cognitive Disorders Unit, Neurology Department, Hospital Clinic, Barcelona, Spain; 25 German Center for Neurodegenerative Diseases, Magdeburg, Germany; 26 Institute of Cognitive Neurology and Dementia Research, Otto-von-Guericke University, Magdeburg, Germany; 27 Technical University of Munich, School of Medicine, Klinikum rechts der Isar, Department of Psychiatry and Psychotherapy, Munich, Germany; 28 Department of Psychiatry and Psychotherapy, University Medical Center Goettingen, Goettingen, Germany; 29 German Center for Neurodegenerative Diseases, Goettingen, Germany; 30 Medical Science Department, Instituto de Biomedicina, Aveiro, Portugal; 31 Institute of Human Genetics, University of Bonn, School of Medicine and University Hospital Bonn, Bonn, Germany; 32 Institute for Urban Public Health, University Hospital of University Duisburg-Essen, Essen, Germany; 33 First Department of Neurology, Medical School, Aristotle University of Thessaloniki, Thessaloniki, Greece; 34 First Department of Neurology, Aiginition Hospital, National and Kapodistrian University of Athens, Medical School, Athens, Greece; 35 Taub Institute for Research in Alzheimer’s Disease and the Aging Brain, The Gertrude H. Sergievsky Center, Department of Neurology, Columbia University, New York, New York; 36 Department of Neurology, II B Sant Pau, Hospital de la Santa Creu i Sant Pau, Universitat Autònoma de Barcelona, Barcelona, Spain; 37 Department of Neurology, Hospital Universitario Donostia, San Sebastian, Spain; 38 Neurosciences Area, Instituto Biodonostia, San Sebastian, Spain; 39 Unitat de Genètica Molecular, Institut de Biomedicina de València, Consejo Superior de Investigaciones Científicas, Valencia, Spain; 40 Unidad Mixta de Neurologia Genètica, Instituto de Investigación Sanitaria La Fe, Valencia, Spain; 41 Centro de Biología Molecular Severo Ochoa, UAM-CSIC, Madrid, Spain; 42 Instituto de Investigacion Sanitaria Hospital la Paz, Madrid, Spain; 43 Unit of Neurodegenerative Diseases, Department of Neurology, University Hospital Germans Trias i Pujol and The Germans Trias i Pujol Research Institute (IGTP) Badalona, Barcelona, Spain; 44 Alzheimer’s Disease and Other Cognitive Disorders Unit, Service of Neurology, Hospital Clínic of Barcelona, Institut d’Investigacions Biomèdiques August Pi i Sunyer, University of Barcelona, Barcelona, Spain; 45 Laboratorio de Genética, Hospital Universitario Central de Asturias, Oviedo, Spain; 46 Instituto de Investigación Sanitaria del Principado de Asturias, Asturias, Spain; 47 Unidad de Trastornos del Movimiento, Servicio de Neurología y Neurofisiología, Instituto de Biomedicina de Sevilla, Hospital Universitario Virgen del Rocío, Consejo Superior de Investigaciones Científicas, Universidad de Sevilla, Seville, Spain; 48 Unidad Clínica de Enfermedades Infecciosas y Microbiología, Hospital Universitario de Valme, Sevilla, Spain; 49 Depatamento de Especialidades Quirúrgicas, Bioquímica e Inmunología, Facultad de Medicina, Universidad de Málaga, Málaga, Spain; 50 Unitat Trastorns Cognitius, Hospital Universitari Santa Maria de Lleida, Lleida, Spain; 51 Institut de Recerca Biomedica de Lleida, Lleida, Spain; 52 Alzheimer Research Center & Memory Clinic, Instituto Andaluz de Neurociencia, Málaga, Spain; 53 Neurology Service, Marqués de Valdecilla University Hospital, University of Cantabria and IDIVAL, Santander, Spain; 54 Institute of Clinical Medicine, Neurology, University of Eastern Finland, Kuopio, Finland; 55 Institute of Biomedicine, University of Eastern Finland, Kuopio, Finland; 56 Faculty of Medicine, University of Lisbon, Portugal; 57 Clinic of Neurology, UH “Alexandrovska,” Medical University–Sofia, Sofia, Bulgaria; 58 Memory Clinic, Department of Neurology, Charles University, Second Faculty of Medicine and Motol University Hospital, Prague, Czech Republic; 59 International Clinical Research Center, St Anne’s University Hospital Brno, Brno, Czech Republic; 60 Genomics of Neurodegenerative Diseases and Aging, Human Genetics, Vrije Universiteit Amsterdam, Amsterdam UMC, locatie VUmc, Amsterdam, the Netherlands; 61 Alzheimer Center Amsterdam, Neurology, Vrije Universiteit Amsterdam, Amsterdam UMC, locatie VUmc, Amsterdam, the Netherlands; 62 Amsterdam Neuroscience, Neurodegeneration, Amsterdam, the Netherlands; 63 Department of Neurobiology, Care Sciences and Society, Division of Neurogeriatrics, Karolinska Institutet, Center for Alzheimer Research, Stockholm, Sweden; 64 Unit for Hereditary Dementias, Theme Aging, Karolinska University Hospital-Solna, Stockholm, Sweden; 65 Aging Research Center, Department of Neurobiology, Care Sciences and Society, Karolinska Institutet and Stockholm University, Stockholm, Sweden; 66 Department of Public Health and Caring Sciences/ and Geriatrics, Uppsala University, Uppsala, Sweden; 67 Université Paris-Saclay, CEA, Centre National de Recherche en Génomique Humaine, Evry, France; 68 Normandie Univ, Université de Rouen Normandie, Inserm U1245 and CHU Rouen, Department of Genetics and Centre national de référence pour les malades Alzheimer jeunes, Rouen, France; 69 inserm, Bordeaux Population Health Research Center, UMR 1219, University of Bordeaux, ISPED, CIC 1401-EC, Bordeaux, France; 70 CHU de Bordeaux, Pole santé publique, Bordeaux, France; 71 University of Lille, Inserm, CHU Lille, UMR1172, Resources and Research Memory Center of Distalz, Licend, Lille, France; 72 Université de Paris, EA 4468, Assistance Publique – Hôpitaux de Paris, Hôpital Broca, Paris, France; 73 University Bordeaux, Inserm, Bordeaux Population Health Research Center, France; 74 Department of Neurology, Bordeaux University Hospital, Bordeaux, France; 75 Department of Child and Adolescent Psychiatry and Psychotherapy, Psychiatric University Hospital Zurich, University of Zurich, Zurich, Switzerland; 76 Neuroscience Center Zurich, University of Zurich and ETH Zurich, Zurich, Switzerland; 77 Zurich Center for Integrative Human Physiology, University of Zurich, Zurich, Switzerland; 78 Old Age Psychiatry, Department of Psychiatry, Lausanne University Hospital, Lausanne, Switzerland; 79 Department of Geriatric Psychiatry, University Hospital of Psychiatry Zürich, Zürich, Switzerland; 80 Department of Psychiatry, Psychotherapy and Psychosomatics, University of Zürich, Zurich, Switzerland; 81 Molecular Markers Laboratory, IRCCS Istituto Centro San Giovanni di Dio Fatebenefratelli, Brescia, Italy; 82 Neurodegenerative Diseases Unit, Fondazione IRCCS Ca’ Granda, Ospedale Policlinico, Milan, Italy; 83 Department of Biomedical, Surgical and Dental Sciences, University of Milan, Milan, Italy; 84 Department of Clinical Sciences and Community Health, University of Milan, Milan, Italy; 85 Geriatric Unit, Fondazione IRCCS Ca’ Granda Ospedale Maggiore Policlinico, Milan, Italy; 86 Institute of Gerontology and Geriatrics, Department of Medicine and Surgery, University of Perugia, Perugia, Italy; 87 Division of Clinical Geriatrics, Department of Neurobiology, Care Sciences and Society, Karolinska Institutet, Stockholm, Sweden; 88 Interdisciplinary Department of Medicine, Geriatric Medicine and Memory Unit, University of Bari Aldo Moro, Bari, Italy; 89 Centre for Memory Disturbances, Lab of Clinical Neurochemistry, Section of Neurology, University of Perugia, Perugia, Italy; 90 Department of Biomedical Sciences, Section of Neuroscience and Clinical Pharmacology, University of Cagliari, Cagliari, Italy; 91 Neurology Unit, Hospital San Gerardo, Monza and University of Milano-Bicocca, Milan, Italy; 92 Centre for Neurodegenerative Disorders, Neurology Unit, Department of Clinical and Experimental Sciences, University of Brescia, Brescia, Italy; 93 Department of Neuroscience, Psychology, Drug Research and Child Health University of Florence, Florence, Italy; 94 IRCCS Fondazione Don Carlo Gnocchi, Florence, Italy; 95 Unit of Neurology, University of Parma, Parma, Italy; 96 Laboratory for Advanced Hematological Diagnostics, Department of Hematology and Stem Cell Transplant, Vito Fazzi Hospital, Lecce, Italy; 97 Department of Neuroscience Rita Levi Montalcini, University of Torino, Torino, Italy; 98 Department of Neuroscience, Università Cattolica del Sacro Cuore, Rome, Italy; 99 Neurology Unit, IRCCS Fondazione Policlinico Universitario A. Gemelli, Rome, Italy; 100 Institute of Neurology, Università Cattolica del Sacro Cuore, Rome, Italy; 101 Laboratory of Neuropsychiatry, IRCCS Santa Lucia Foundation, Rome, Italy; 102 Department of Psychiatry and Behavioral Sciences, Baylor College of Medicine, Houston, Texas; 103 UK Dementia Research Institute at Cardiff, School of Medicine, Cardiff University, Cardiff, United Kingdom; 104 Cluster of Excellence Cellular Stress Responses in Aging-Associated Diseases, University of Cologne, Cologne, Germany; 105 Translational Health Sciences, Bristol Medical School, University of Bristol, Bristol, United Kingdom; 106 Alzheimer Hellas, Thessaloniki, Greece; 107 Unit of Neurology V - Neuropathology, Fondazione IRCCS Istituto Neurologico Carlo Besta, Milan, Italy; 108 Alzheimer’s Centre Reina Sofia-CIEN Foundation-ISCIII, Madrid, Spain; 109 Department of Biomedical Sciences, University of Antwerp, Antwerp, Belgium; 110 Krembil Brain Institute, University Health Network, Toronto, Canada; 111 Department of Medicine and Tanz Centre for Research in Neurodegenerative Diseases, University of Toronto, Toronto, Canada; 112 NORMENT Centre, Division of Mental Health and Addiction, Oslo University Hospital, Oslo, Norway; 113 Institute of Clinical Medicine, University of Oslo, Oslo, Norway; 114 Department of Epidemiology, ErasmusMC, Rotterdam, the Netherlands; 115 Nuffield Department of Population Health, Oxford University, Oxford, United Kingdom; 116 Department of Psychiatry and Glenn Biggs Institute for Alzheimer’s and Neurodegenerative Diseases, San Antonio, Texas; 117 Department of Clinical Medicine, University of Copenhagen, Copenhagen, Denmark

## Abstract

**Importance:**

An estimated 40% of dementia is potentially preventable by modifying 12 risk factors throughout the life course. However, robust evidence for most of these risk factors is lacking. Effective interventions should target risk factors in the causal pathway to dementia.

**Objective:**

To comprehensively disentangle potentially causal aspects of modifiable risk factors for Alzheimer disease (AD) to inspire new drug targeting and improved prevention.

**Design, Setting, and Participants:**

This genetic association study was conducted using 2-sample univariable and multivariable mendelian randomization. Independent genetic variants associated with modifiable risk factors were selected as instrumental variables from genomic consortia. Outcome data for AD were obtained from the European Alzheimer & Dementia Biobank (EADB), generated on August 31, 2021. Main analyses were conducted using the EADB clinically diagnosed end point data. All analyses were performed between April 12 and October 27, 2022.

**Exposures:**

Genetically determined modifiable risk factors.

**Main Outcomes and Measures:**

Odds ratios (ORs) and 95% CIs for AD were calculated per 1-unit change of genetically determined risk factors.

**Results:**

The EADB-diagnosed cohort included 39 106 participants with clinically diagnosed AD and 401 577 control participants without AD. The mean age ranged from 72 to 83 years for participants with AD and 51 to 80 years for control participants. Among participants with AD, 54% to 75% were female, and among control participants, 48% to 60% were female. Genetically determined high-density lipoprotein (HDL) cholesterol concentrations were associated with increased odds of AD (OR per 1-SD increase, 1.10 [95% CI, 1.05-1.16]). Genetically determined high systolic blood pressure was associated with increased risk of AD after adjusting for diastolic blood pressure (OR per 10–mm Hg increase, 1.22 [95% CI, 1.02-1.46]). In a second analysis to minimize bias due to sample overlap, the entire UK Biobank was excluded from the EADB consortium; odds for AD were similar for HDL cholesterol (OR per 1-SD unit increase, 1.08 [95% CI, 1.02-1.15]) and systolic blood pressure after adjusting for diastolic blood pressure (OR per 10–mm Hg increase, 1.23 [95% CI, 1.01-1.50]).

**Conclusions and Relevance:**

This genetic association study found novel genetic associations between high HDL cholesterol concentrations and high systolic blood pressure with higher risk of AD. These findings may inspire new drug targeting and improved prevention implementation.

## Key Points

**Question:**

What are the genetic associations between modifiable risk factors and Alzheimer disease (AD)?

**Findings:**

In this genetic association study using a mendelian randomization framework with the largest genomic data sets to date, including 39 106 participants with clinically diagnosed AD and 401 577 control participants without AD, genetically determined increased high-density lipoprotein cholesterol and increased systolic blood pressure were associated with higher risk of AD.

**Meaning:**

These findings suggest that genetically determined increased high-density lipoprotein cholesterol and systolic blood pressure may be involved in AD pathogenesis, which may thus inspire new drug targeting and improved early dementia prevention.

## Teaser

This genetic association study uses mendelian randomization to examine potentially causal aspects of modifiable genetic risk factors for Alzheimer disease.

## Introduction

Dementia is a rapidly increasing health threat worldwide, affecting more than 50 million people and projected to triple in prevalence by 2050.^1^ A 2020 report by Livingston et al for the *Lancet Commission for Dementia Prevention, Intervention and Care*^1^ estimated that up to 40% of dementia could be prevented or delayed by modifying 12 risk factors throughout the life course.^1^ However, various degrees of inconsistency for these risk factors exist between observational studies and clinical trials, leading to mixed quality of evidence underpinning recommendations.^2,3^ Effective interventions should target ameliorating risk factors that lie in the causal pathways. Hence, thoroughly unfolding the genomic background for associations between modifiable risk factors and dementia might help to develop future efficacious preventive and therapeutic approaches.

Associations identified in observational studies are not equivalent to causality due to confounding and reverse causation; the latter may explain why associations between risk factors and dementia change across the lifespan, especially in late life.^4,5^ Although randomized clinical trials may demonstrate an unconfounded effect of a certain intervention on dementia, interventions after irreversible neuron damage or with a relatively short duration may have a negligible effect. The mendelian randomization (MR) design uses genetic variants associated with the exposure to investigate potential causal relationships between risk factors and outcomes. The exposure is thus lifelong, and the random allocation of variants at conception minimizes confounding and reverse causation.^6^ Thus, the MR approach may help establish causality and guide whether a comprehensive randomized clinical trial targeting the risk factor will be meaningful to perform. Several MR studies have been conducted to disentangle the associations between modifiable risk factors and Alzheimer disease (AD), the most common type of dementia and the only type of dementia with large-scale genomewide association studies (GWAS). Genetically, longer educational attainment has been well-established as associated with lower risk for AD, whereas other risk factors, including lipid traits, blood pressure (BP), body mass index (BMI), smoking, and alcohol consumption have shown inconclusive associations with AD. This may be due to lack of power, small number of genetic instruments, and other biases related to study design. Consequently, more powerful and state-of-the-art MR studies are warranted to examine genomic associations between modifiable risk factors and AD.

The new landmark paper on AD genetic etiology by the European Alzheimer & Dementia Biobank (EADB) provides new possibilities to disentangle potential causal aspects of modifiable risk factors for AD.^7^ Furthermore, the massively increasing availability of high-quality genotypic data in large consortia provides more powerful genetic instrumental variables. Collectively, this prompted us to scrutinize the genetic associations between modifiable risk factors and AD using complementary and up-to-date MR methods.

## Methods

**Figure 1.**
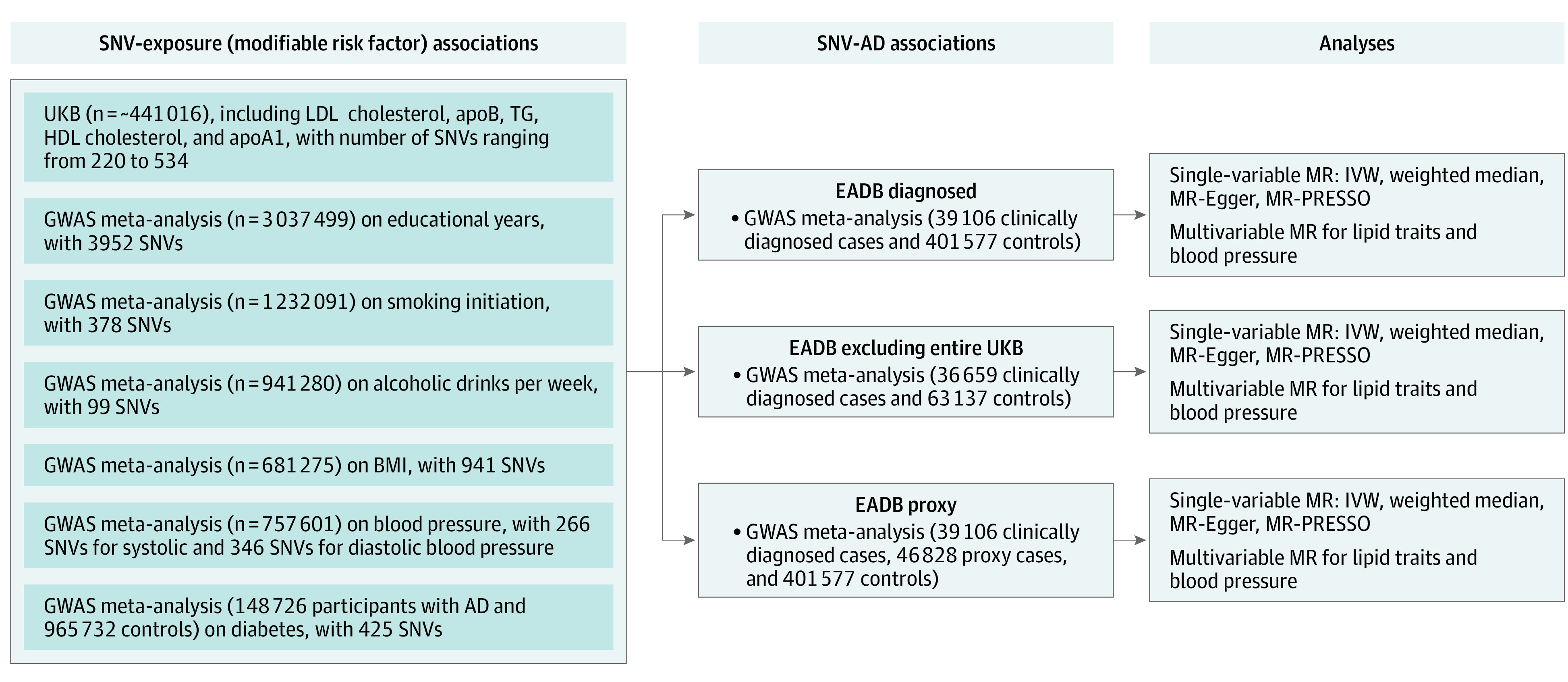
Schematic Overview of the Study Design AD indicates Alzheimer disease; apoA1, apolipoprotein A1; apoB, apolipoprotein B; BMI, body mass index; EADB, European Alzheimer & Dementia Biobank; GWAS, genome-wide association study; HDL, high-density lipoprotein; IVW, inverse-variance weighted; LDL, low-density lipoprotein; MR, mendelian randomization; MR-PRESSO, mendelian randomization pleiotropy residual sum and outlier; SNV, single nucleotide variant; TG, triglycerides; UKB, UK Biobank.

In this genetic association study, we implemented a 2-sample MR approach that uses genetic variants as instrumental variables for the exposure to investigate whether a lifetime exposure may be causally associated with an outcome (eFigure 1 in Supplement 1). Ethical approvals were obtained by each individual participating cohort; therefore, no additional ethical approvals or informed consents were required. Our study followed the Strengthening the Reporting of Genetic Association Studies (STREGA) reporting guideline and Strengthening the Reporting of Observational Studies in Epidemiology Using Mendelian Randomization (STROBE-MR) reporting guidelines. The schematic overview of the study design is presented in Figure 1, and previous main MR studies on modifiable risk factors and AD are listed in eTable 1 in Supplement 1. We used summary GWAS statistics for each exposure and AD.

### Selection of Instrumental Variables

Modifiable risk factors include educational attainment,^8^ lipids and lipoproteins^9^ (low-density lipoprotein [LDL] cholesterol, triglycerides [TG], apolipoprotein B [apoB], high-density lipoprotein [HDL] cholesterol, and apolipoprotein A1 [apoA1]), BMI,^10^ alcohol consumption,^11^ smoking initiation,^11^ systolic BP (SBP) and diastolic BP (DBP),^12^ and type 2 diabetes.^13^ Details on the GWAS from which we obtained summary-level associations between genetic variants and risk factors are described in Table 1. Selection and summary information of the independent single nucleotide variants (SNVs) are in Table 2 and the eMethods in Supplement 1.

**Table 1.** GWAS Data Sources for Instrumental Variables Selection

Study	Risk factor	Consortium	Participants^a^	Covariates
Okbay et al,^8^ 2022	Education attainment	SSGAC; UKB; 23andMe	3 037 499	Age, sex, age × sex, PCs
Richardson et al,^9^ 2020	Low-density lipoprotein cholesterol	UKB	440 546	Age, sex
	High-density lipoprotein cholesterol		403 943	
	Triglycerides		441 016	
	Apolipoprotein A1		393 193	
	Apolipoprotein B		439 214	
Yengo et al,^10^ 2018	BMI	GIANT, UKB	681 275	Age, sex, PCs
Liu et al,^11^ 2019	Smoking initiation	GSCAN; 23andMe	1 232 091	Age, sex, age × sex, PCs
	Alcohol consumption		941 280	
Evangelou et al,^12^ 2018	Systolic blood pressure	UKB; ICBP	757 601	Age, sex, age^2^, BMI
	Diastolic blood pressure			
Vujkovic et al,^13^ 2020	Type 2 diabetes	DIAMENTE	148 726 with AD	Age, sex, PCs
			965 732 controls	

Abbreviations: AD, Alzheimer disease; BMI, body mass index; DIAMENTE, Diabetes Meta-analysis of Trans-ethnic Association Studies consortium; GIANT, Genetic Investigation of Anthropometric Traits consortium; GWAS, genome-wide association study; GSCAN, GWAS and Sequencing Consortium of Alcohol and Nicotine use; ICBP, International Consortium of Blood Pressure Genome Wide Association Studies; PCs, principal components; SSGAC, Social Science Genetic Association Consortium; UKB, UK Biobank.

^a^ Numbers are participants with European ancestry.

**Table 2.** Summary Information of Genetic Instruments for Modifiable Risk Factors

Risk factor	Unit	SNVs, No.^a^	LD threshold^b^	Variation, %
Low-density lipoprotein cholesterol	SD in mmol/L	220	0.001	7.7^c^
Triglycerides	SD in mmol/L	440	0.001	10.3^c^
Apolipoprotein B	SD in g/L	255	0.001	9.2^c^
High-density lipoprotein cholesterol	SD in mmol/L	534	0.001	11.9^c^
Apolipoprotein A1	SD in g/L	440	0.001	10.1^c^
Educational attainment	years	3952	0.1	12-16
BMI	SD per 1 unit	507	0.001	6
Smoking initiation	Ever smoked regularly vs never smoke	378	0.1	2.3
Alcohol consumption	SD in alcoholic drinks per week	99	0.1	0.7
Systolic blood pressure	10 mm Hg	266	0.1	5.7
Diastolic blood pressure	10 mm Hg	346	0.1	5.3
Type 2 diabetes	Log odds	425	0.05	19

Abbreviations: BMI, body mass index (calculated as weight in kilograms divided by height in meters squared); LD, linkage disequilibrium; SNV, single nucleotide variant.

^a^ Number of independent SNVs at genome-wide significance level (*P* < 5 × 10^−8^).

^b^ LD refers to the degree to which an allele of 1 genetic variant is inherited or correlated with an allele of a nearby genetic variant within a given population. The threshold to prune for LD was obtained in the original genome-wide association studies.

^c^ Variation explained by genetic instrumental variables were calculated based on the formula: β^2^ × 2 × *MAF* × (1 − *MAF*), where *MAF* denotes mean minor allele frequency from European populations, obtained through Phenoscanner V2. Calculation of the remaining percentages are given in the original articles.

### Alzheimer Disease Data Sources

The associations between SNVs and late-onset AD were retrieved from EADB, the largest AD genomic consortia. EADB brings together a range of European cohorts and GWAS consortia, and summary estimates were based on 39 106 participants with clinically diagnosed AD, 46 828 participants with proxy AD, and 401 577 control participants without AD^7^ (generated in August 31, 2021). Proxy AD was only identified from the UK Biobank via questionnaire data asking if parents of the participants had AD (“Has/did your father or mother ever suffer from Alzheimer’s disease/dementia?”). Participants were categorized into proxy AD if the answer was yes, otherwise they were controls. Three summary data sets were used: (1) the EADB-diagnosed data set in which only participants who had been clinically diagnosed with AD were included in the summary data; (2) the EADB data set, excluding the entire UK Biobank (UKB) (generated on September 19, 2022); and (3) the EADB-proxy data set, which included both participants who had been clinically diagnosed with AD and those with proxy AD (eMethods in Supplement 1) from the UKB were included (generated on February 10, 2022).

### Statistical Analysis

All analyses were performed between April 12 to October 27, 2022. The MR results are given as odds ratios (ORs) with corresponding 95% CIs of log odds of AD per unit increase in genetically determined risk factors. The estimates are scaled by year of education completed, ever smoked regularly vs never smoked, 10–mm Hg increase of BP, SD for consumption of alcoholic drinks per week, and SD for the other continuous risk factors; for diabetes, the estimates represent the OR of AD per 1-unit higher log odds of diabetes. In the reverse direction, the results represent the relative increase in the odds of AD per 1-unit change in each behavioral risk factor. Statistical power for MR analyses were calculated using the power calculation tool.^14^ We have 80% power to detect a minimum of 3% change in log odds of AD (eFigure 2 in Supplement 1).

To maintain statistical power while still limiting the number of false-positive conclusions, we corrected for multiple testing per MR-method using false discovery rate proposed by Benjamini and Hochberg.^15^ Two-sided *P* < .05 indicated statistical significance. All the analyses were undertaken using R version 4.0.2 (R Project for Statistical Computing).

Associations between genetic variants and risk factors and AD were harmonized to ensure that estimates were aligned on the same allele. Ambiguous genetic variants with palindromic genotypes were excluded. We used the inverse-variance weighted (IVW) method as the primary analysis, which combines SNV-specific estimates calculated by Wald ratios through dividing the genetic association with AD by the genetic association with each risk factor. When a genetic variant affects other traits that influence the outcome independently of the hypothesized exposure, known as horizontal pleiotropy, this may violate 1 of the key MR assumptions of exclusion restriction. IVW assumes no violation of MR assumptions, particularly no directional pleiotropic effect of each instrumental variable, and constrains intercepts to zero. Furthermore, we performed several MR sensitivity analyses to address invalid instruments, unbalanced pleiotropy, outliers, and correlated risk factors. The weighted median estimator and MR-Egger allows the inclusion of pleiotropic genetic variants and were used to investigate whether bias in IVW estimates were present due to invalid instruments.^16,17^ The regression slope from MR-Egger represents the estimated effect of an exposure on the outcome, and the freely estimated intercept additionally provides a mean magnitude of the pleiotropic effects across all genetic variants if it deviates from zero. MR-Egger is statistically less efficient (ie, with wider CIs) but provides a causal estimate that accounts for horizontal pleiotropy. Therefore, the point estimates from these 2 methods might be close to null even if a strong association is observed through IVW; however, the CIs should largely overlap. To assess the distortions of the IVW estimate from any heterogeneity or horizontal pleiotropy, MR-PRESSO was applied to detect and correct for outliers, giving an unbiased estimate.^18^

For correlated risk factors, we performed multivariable MR, an extension of the basic MR design and estimates the effects of 2 or more related exposures on an outcome simultaneously. Subsequently, the direct effect, ie, the effect not confounded or mediated by other factors, of each exposure in the model is obtained.^19^ For lipids, apoA1 and apoB are highly correlated with HDL and LDL cholesterol, respectively. To avoid multicollinearity in the multivariable MR model, we adjusted HDL and TG for LDL and apoB, HDL and LDL for TG, and LDL and TG for HDL and apoA1.

Four additional sensitivity analyses were conducted and are described in detail in eMethods in Supplement 1. First, we used the Causal Analysis using Summary Effect Estimates) method, accounting for correlated pleiotropy.^20^ A second analysis excluded SNVs on the entire chromosome 19, addressing the independence of the strong apolipoprotein E (*APOE*) locus. Third, we used cross-trait linkage disequilibrium-score regression, accounting for sample overlap.^21^ Fourth, we evaluated possible reverse causation of AD on behavioral risk factors.

## Results

### EADB-Diagnosed Data Set

The EADB-diagnosed cohort included 39 106 participants with clinically diagnosed AD and 401 577 control participants without AD. In the EADB-diagnosed data set, the mean age ranged from 72 to 83 years among participants with AD and 51 to 80 years among control participants without AD. Among participants with AD, 54% to 75% were female, and among control participants, 48% to 60% were female. A detailed description of the demographic characteristic is given in the original literature.^7^

**Figure 2.**
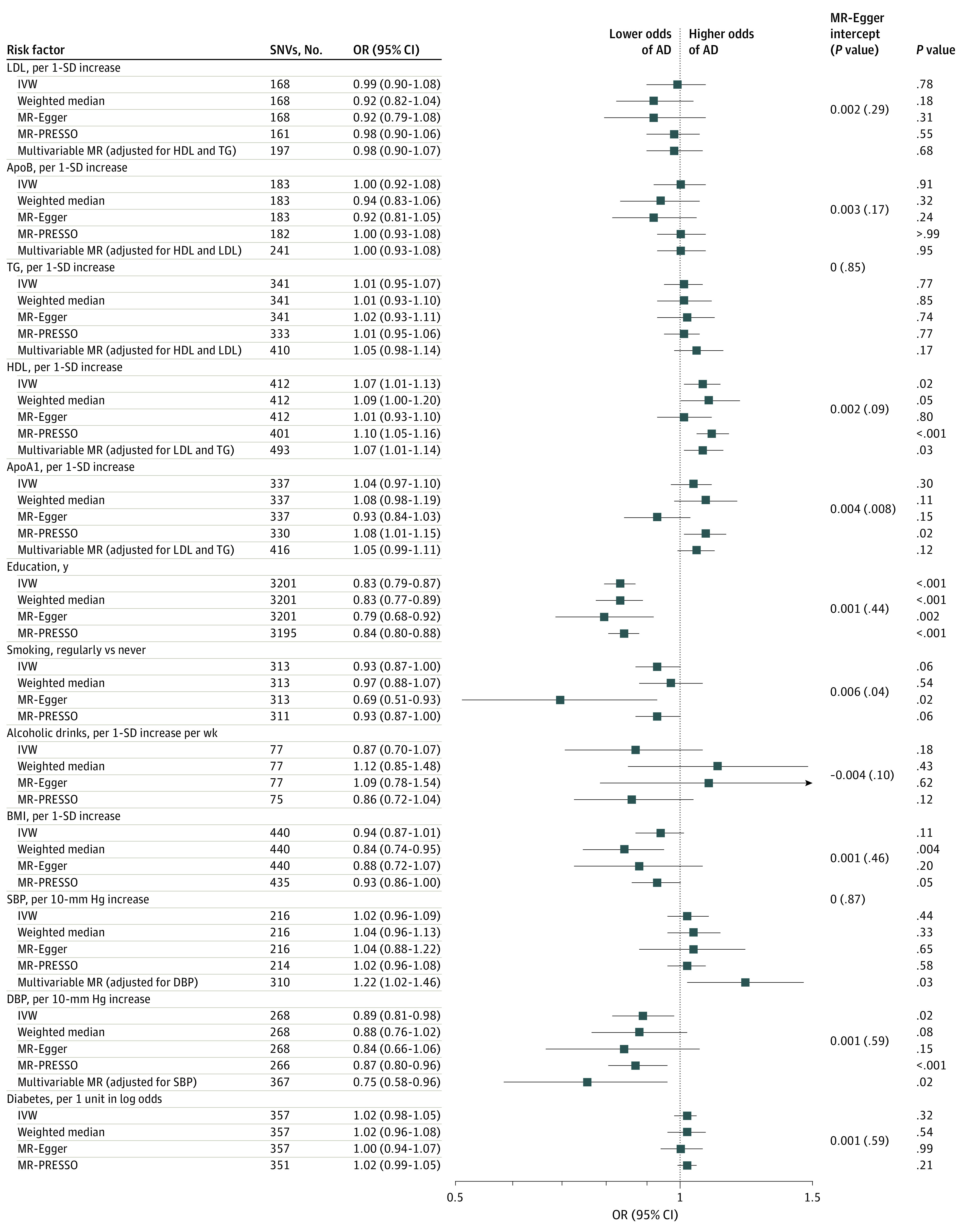
Associations of Genetically Determined Modifiable Risk Factors and Alzheimer Disease (AD) in the European Alzheimer & Dementia Biobank Data Set of Participants With Clinically Diagnosed AD Multivariable mendelian randomization was performed for correlated phenotypes only (lipid traits and blood pressure). ApoA1 indicates apolipoprotein A1; apoB, apolipoprotein B; BMI, body mass index; DBP, diastolic blood pressure; HDL, high-density lipoprotein; IVW, inverse-variance weighted; LDL, low-density lipoprotein; MR-PRESSO, mendelian randomization pleiotropy residual sum and outlier; OR, odds ratio; SBP, systolic blood pressure; SNV, single nucleotide variant; TG, triglycerides.

The results from the EADB-diagnosed data set are presented in eTable 1 in Supplement 2 and visualized in Figure 2. Increased HDL cholesterol was associated with increased odds of AD (OR per 1-SD increase, 1.07 [95% CI, 1.01-1.13]). The point estimate for HDL cholesterol was enhanced on correction for outliers using MR-PRESSO (OR, 1.10 [95% CI, 1.05-1.16]; *P* < .001) and remained similar to the IVW estimate in other MR sensitivity methods (eTable 1 in Supplement 2). After adjusting for DBP, SBP was associated with increased risk of AD in multivariable MR (OR per 10–mm Hg increase, 1.22 [95% CI, 1.02-1.46]). Analyses using different univariable MR methods were not statistically significant (OR per 10–mm Hg increase, 1.02 [95% CI, 0.96-1.09]), but analysis using other MR sensitivity methods remained significant. A 10–mm Hg genetically determined higher level of DBP was associated with lower risk of AD across different MR methods. Genetic predisposition to longer educational attainment was associated with lower odds of AD in all analyses (IVW OR, 0.83 [95% CI, 0.79-0.87]). The estimates for apoA1, smoking, and BMI were inconclusive. LDL cholesterol, apoB, TG, alcohol consumption, and diabetes were consistently not associated with the odds of AD in all MR methods. A detailed list of SNVs involved in the HDL cholesterol signal is given in eTable 2 in Supplement 2.

### EADB Excluding the Entire UKB

**Figure 3.**
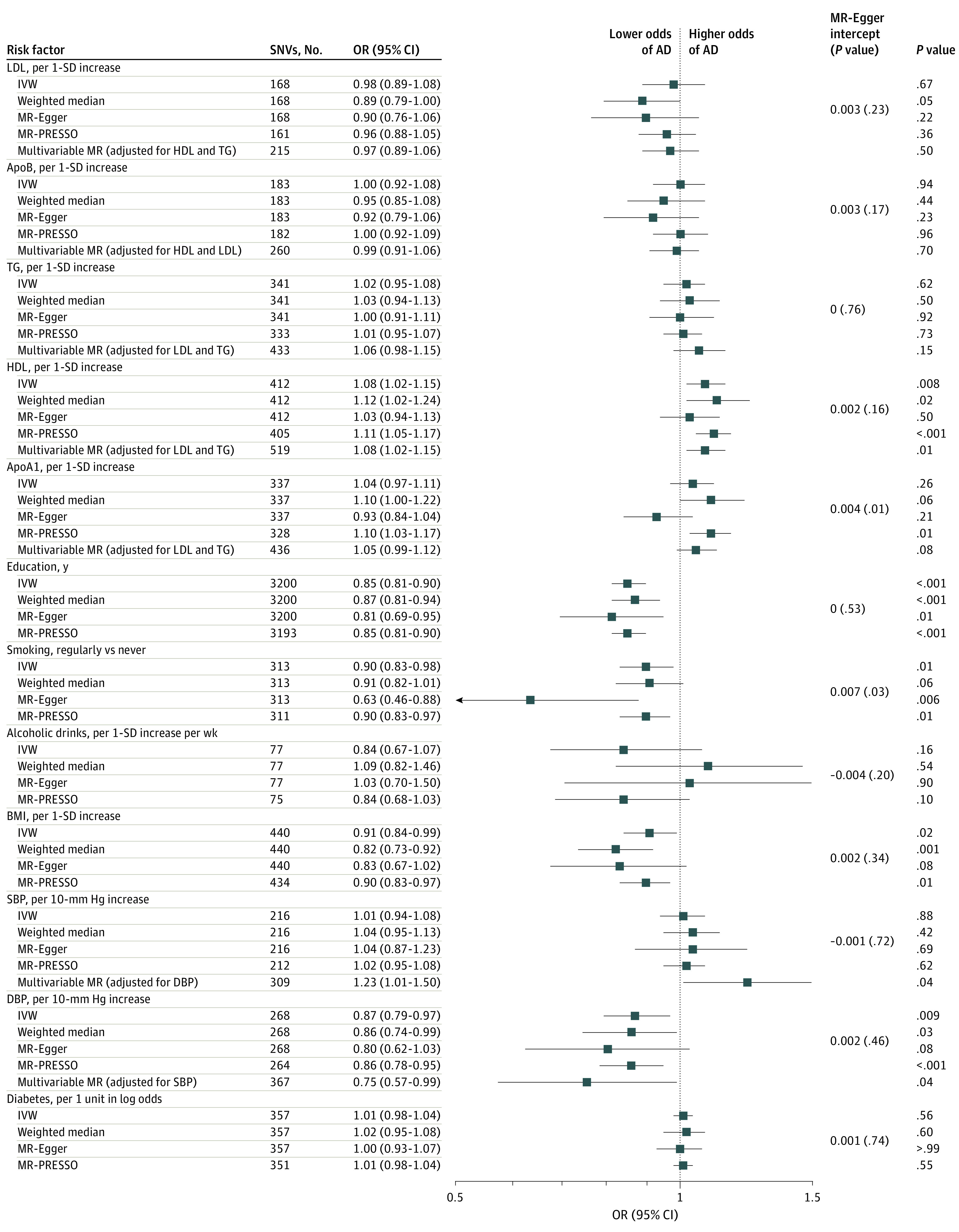
Associations of Genetically Determined Modifiable Risk Factors and Alzheimer Disease (AD) in the European Alzheimer & Dementia Biobank Data Set Excluding the Entire UK Biobank Multivariable mendelian randomization was performed for correlated phenotypes only (lipid traits and blood pressure). Abbreviations: apoA1, apolipoprotein A1; apoB, apolipoprotein B; BMI, body mass index; DBP, diastolic blood pressure; HDL, high-density lipoprotein; IVW, inverse-variance weighted; LDL, low-density lipoprotein cholesterol; MR-PRESSO, mendelian randomization pleiotropy residual sum and outlier; OR, odds ratio; SBP, systolic blood pressure; SNV, single nucleotide variant; TG, triglycerides.

The results from the EADB data set excluding UKB generally resemble those from the EADB diagnosed data set (Figure 3; eTable 3 in Supplement 2). For HDL cholesterol concentrations, the IVW and multivariable MR analyses found the same results (ORs for both, 1.08 [95% CI, 1.02-1.15]). After adjusting for DBP, increased SBP was associated with increased risk of AD (OR per 10–mm Hg increase, 1.23 [95% CI, 1.01-1.50]). The analyses corrected for multiple testing remained significant in both IVW and MR-PRESSO. Higher DBP was associated with lower risk of AD in the IVW analysis (OR per 10–mm Hg increase, 0.87 [95% CI, 0.79-0.97]) and remained similar using other sensitivity methods. Genetic predisposition to longer educational attainment was associated with lower odds of AD in all analyses (IVW OR, 0.85 [95% CI, 0.81-0.90]). Smoking initiation and higher BMI were associated with lower odds of AD (smoking: IVW OR, 0.90 [95% CI, 0.83-0.98]; BMI: IVW OR, 0.91 [95% CI, 0.84-0.99]). No associations were found between other modifiable risk factors and odds of AD.

### EADB-Proxy Data Set

Results using EADB-proxy data set are shown in eFigure 3 in Supplement 1. Longer educational attainment was associated with higher odds of AD (IVW OR, 1.06 [95% CI, 1.01-1.10]). A detailed illustration leading to this counterintuitive finding is provided in eFigure 4 in Supplement 1. In IVW analyses, higher HDL cholesterol (OR, 1.10 [95% CI, 1.04-1.15]) and higher apoA1 (OR, 1.07 [95% CI, 1.00-1.13]) were associated with higher odds of AD, whereas smoking (OR, 0.88 [95% CI, 0.82-0.94]), higher BMI (OR, 0.89 [95% CI, 0.83-0.95]), and higher DBP (OR, 0.85 [95% CI, 0.78-0.92]) were associated with lower odds of AD. LDL, apoB TG, alcohol consumption, SBP, and diabetes showed no association (eFigure 3 in Supplement 1).

### Other Sensitivity Analyses

The various sensitivity analyses examining the association between genetically determined modifiable risk factors and AD generally showed similar results (eAppendix 1, eTable 2, eFigure 5, and eFigure 6 in Supplement 1). Furthermore, genetic predisposition to high odds of AD were not associated with educational attainment, smoking, alcohol consumption, or BMI (eTable 3 in Supplement 1).

## Discussion

This genetic association study using 2-sample mendelian randomization based on the largest genomic consortia found that genetically determined high HDL cholesterol and high SBP were associated with higher odds of AD. There was no consistent evidence supporting genetic associations of other lipid traits, BMI, alcohol consumption, smoking initiation, or diabetes with odds of AD. Moreover, our study suggested that meticulous care should be taken when using individuals with proxy AD from the UKB in 2-sample MR studies, as the results can be seriously biased.

Conflicting results for modifiable risk factors and AD have been reported in previous MR studies. For HDL cholesterol in particular, genetic studies have found no association^22,23,24,25,26,27,28,29^ or an association of high concentration of extra-large particles with lower risk of AD.^30,31^ These inconsistencies may be attributed to insufficient power and other biases, including pleiotropy. To our knowledge, our study is the first to identify an association between high HDL cholesterol concentrations and higher AD risk in a comprehensive range of complementary analyses. The genetic instruments for HDL cholesterol are marking well-known genes in HDL cholesterol biology, including ATP binding cassette A and G transporters, cholesteryl ester transfer protein, endothelial lipase, hepatic lipase, lipoprotein lipase, and scavenger receptor B1, further strengthening the validity of our findings. Although the underlying mechanisms remain unclear, there are a few biologically plausible explanations. HDL particles are complex, comprising a wide spectrum of sizes, compositions, and functionality. Small, but not large, HDL particles exchange lipids between plasma and cerebrospinal fluid compartments and form apoE and apoA1 small HDL particles through the interaction between plasma-derived apoA1 and brain-derived apoE.^32^ These particles subsequently promote neuronal membrane lipid remodeling and synaptic plasticity, limit apoE self-aggregation, and increase receptor binding and amyloid-β clearance.^33^ Indeed, the concentration of small particles in cerebrospinal fluid is highly correlated with the concentrations in plasma and is positively associated with cognitive function.^34^ However, high HDL cholesterol concentrations in plasma lead to a shift toward large HDL particles and significant increases in apoA1.^35^ Therefore, high plasma HDL cholesterol concentrations characterizing large buoyant HDL particles may play a role in dementia pathogenesis by disrupting the homeostasis between plasmatic particles and the beneficial apoE and apoA1 small HDL particles in cerebrospinal fluid.

Observationally, hypertension in midlife has been suggested as an independent risk factor for AD, whereas hypertension in late life showed null or reverse associations with AD, particularly for DBP.^36,37^ Sustained hypertension from midlife to late life, compared with midlife and late-life BP within reference ranges, was associated with increased risk of dementia.^38^ Nevertheless, most studies have BP measured at 1 or few separate time points, which may not fully capture the longitudinal changes and their cumulative effect. Results from individual antihypertensive randomized clinical trials are inconclusive. A meta-analysis combining 12 trials (baseline BP 154/83.3 mm Hg) concluded that antihypertensive treatment is associated with significantly decreased dementia risk through decreasing SBP.^39^ Similarly, a pooled individual-participant data analysis of 5 randomized clinical trials provided evidence supporting benefits associated with antihypertensive treatment in late midlife and later life to lower the risk of dementia.^40^ However, these effects last only for the duration of the trials. Our findings of genetically determined and thus lifelong high SBP and low DBP independently associated with high AD risk were partly in line with previous MR findings.^41,42^ These associations are reinforced by a study in which a long-term cumulative SBP increase was associated with subsequently higher dementia risk, whereas a cumulative DBP increase was associated with lower risk.^43^ There are several hypothesized explanations, which are discussed in detail in eAppendix 2 in Supplement 1.

We observed associations of high BMI and smoking initiation with lower risk of AD. Individual-level data analyses have suggested that the BMI association might be restricted to older age groups only.^44^ The association between smoking initiation or lifetime smoking and AD were mixed in previous MR studies using summary data; individual-level data from a genetically homogenous Danish population in a 1-sample MR study observed a higher risk of AD with high smoking quantity.^45^ Nevertheless, a meta-analysis that pooled the results from 2 summary statistics-based MR studies found no associations either for smoking initiation or quantity^46^; however, the estimates from the included studies showed opposite directions, resulting in significant heterogeneity. The mechanisms behind these findings need further investigation. Finally, no genetic associations of LDL cholesterol, apoB, TG, alcohol consumption, or diabetes with risk of AD were observed.

Despite the confirmation of the association between longer educational attainment and low AD risk in EADB participants with clinically diagnosed AD, the association counterintuitively reversed when including participants with proxy AD. There are significant differences and genetic heterogeneity in the associations between education and clinically diagnosed AD and a self-reported proxy phenotype.^47^ A possible explanation may be that the status of a proxy AD diagnosis may be associated with the person’s educational level, which most likely is influenced by their parental educational level. Thus, the selected instrumental variables were associated with parental educational attainment through the genetic variants for parental education, violating the independence assumption of the MR design. This may also apply to other modifiable factors that are associated with education, such as LDL cholesterol and BMI, as manifested by their associations with AD becoming stronger when including proxy AD diagnoses.

The main strength of this study is the use of the largest genomic consortia to date, yielding ample statistical power and instrumental variables explaining much phenotypic variation. The mixed definition of AD in the consortia allowed us to explore the potential influence of proxy AD on the associations between behavioral risk factors and AD. Furthermore, several MR sensitivity analyses were performed to account for bias related to study design. The statistical correction for sample overlap between exposure and end point data, as well as the possibility to use the EADB data excluding the entire UKB, enabled us to produce robust findings.

### Limitations

This study has some limitations. An inherent limitation of our study is that the included genetic studies predominantly consist of individuals of European ancestry, which limits the extrapolation of our findings to individuals of other ethnicities. Moreover, to our knowledge, all MR studies performed to date take advantage of genetic variants that are associated with 1-time measurement of the exposure. The associations of some risk factors at midlife and late life, such as BMI, have been contradictory. This may also explain the associations of high BMI with lower AD risk both in previous MR studies and in our MR study. Several observational studies have examined risk factor trajectories throughout the life course, capturing a more complete picture and representing the associations of time-varying factors, which might be more relevant than a single point measurement in examining risk of AD. However, no additional analyses could be performed due to the lack of trajectory GWAS.

## Conclusions

This genetic association study found novel genetic associations between high HDL cholesterol concentrations and high SBP with higher risk of AD. These findings may inspire new drug targeting and improved early dementia prevention.

## Notes

Supplement 1.
eMethods.
**eAppendix 1.** Supplementary Results **eAppendix 2.** Supplementary Discussion **eFigure 1.** The Concept of Mendelian Randomization Design **eFigure 2.** Statistical Power of Each Exposure in the EADB Consortium **eFigure 3.** Associations of Genetically Determined Modifiable Risk Factors and AD in the EADB-Proxy Data Set **eFigure 4.** Directed Acyclic Graph Illustration on the Association Between Educational Attainment and Proxy-AD **eFigure 5.** Associations of Genetically Determined Modifiable Risk Factors and AD in the EADB-Diagnosed Data Set Excluding SNVs From Chromosome 19 **eFigure 6.** Associations of Genetically Determined Modifiable Risk Factors and AD in the EADB-Proxy Data Set Excluding SNVs From Chromosome 19 **eTable 1. **Previous Main Mendelian Randomization Studies on Modifiable Risk Factors and AD Based on Summary Statistics **eTable 2.** Results From CAUSE Mendelian Randomization **eTable 3.** Association of Genetic Predisposition to High Risk of AD and Behavioral Risk Factors **eAppendix 3.** Acknowledgements for EADB Cohorts
eReferences

Supplement 2. **eTable 1.** Mendelian Randomization Results From EABD-Diagnosed Data Set **eTable 2.** Information of Genetic Instrumental Variables for High-Density Lipoprotein Cholesterol **eTable 3.** Mendelian Randomization Results From EADB Dataset Excluding the Entire UKB

Supplement 3.
Data Sharing Statement
