# Errors in Author Affiliations

**DOI:** 10.1001/jamanetworkopen.2023.21189

**Published:** 2023-06-05

In the Original Investigation titled “Genetic Associations Between Modifiable Risk Factors and Alzheimer Disease,”^1^ published May 17, 2023, there were errors in the name of Dr Tsolaki and in the affiliations listed for Drs Graff, Tsolaki, Ingelsson, Hiltunen, and van der Flier. This article has been corrected.^1^
