# Supplementary material for: Genetic Associations Between Modifiable Risk Factors and Alzheimer Disease
Source: JAMA Netw Open. 2023 May 17;6(5):e2313734. doi: 10.1001/jamanetworkopen.2023.13734 (PMC10193187; doi:10.1001/jamanetworkopen.2023.13734)
Supplement: Supplement 1. — eMethods. eAppendix 1. Supplementary Results eAppendix 2. Supplementary Discussion eFigure 1. The Concept of Mendelian Randomization Design eFigure 2. Statistical Power of Each Exposure in the EADB Consortium eFigure 3. Associations of Genetically Determined Modifiable Risk Factors and AD in the EADB-Proxy Data Set eFigure 4. Directed Acyclic Graph Illustration on the Association Between Educational Attainment and Proxy-AD eFigure 5. Associations of Genetically Determined Modifiable Risk Factors and AD in the EADB-Diagnosed Data Set Excluding SNVs From Chromosome 19 eFigure 6. Associations of Genetically Determined Modifiable Risk Factors and AD in the EADB-Proxy Data Set Excluding SNVs From Chromosome 19 eTable 1. Previous Main Mendelian Randomization Studies on Modifiable Risk Factors and AD Based on Summary Statistics eTable 2. Results From CAUSE Mendelian Randomization eTable 3. Association of Genetic Predisposition to High Risk of AD and Behavioral Risk Factors eAppendix 3. Acknowledgements for EADB Cohorts eReferences [file jamanetwopen-e2313734-s001.pdf]

Supplemental Online Content

European Alzheimer’s & Dementia Biobank Mendelian Randomization (EADB-MR) Collaboration. Genetic associations between modifiable risk factors and Alzheimer disease. *JAMA Netw Open*. 2023;6(5):e2313734. doi:10.1001/jamanetworkopen.2023.13734

eMethods.

eAppendix 1. Supplementary Results

eAppendix 2. Supplementary Discussion

eFigure 1. The Concept of Mendelian Randomization Design

eFigure 2. Statistical Power of Each Exposure in the EADB Consortium

eFigure 3. Associations of Genetically Determined Modifiable Risk Factors and AD in the EADB-Proxy Data Set

eFigure 4. Directed Acyclic Graph Illustration on the Association Between Educational Attainment and Proxy-AD

eFigure 5. Associations of Genetically Determined Modifiable Risk Factors and AD in the EADB-Diagnosed Data Set Excluding SNVs From Chromosome 19

eFigure 6. Associations of Genetically Determined Modifiable Risk Factors and AD in the EADB-Proxy Data Set Excluding SNVs From Chromosome 19

eTable 1. Previous Main Mendelian Randomization Studies on Modifiable Risk Factors and AD Based on Summary Statistics

eTable 2. Results From CAUSE Mendelian Randomization

eTable 3. Association of Genetic Predisposition to High Risk of AD and Behavioral Risk Factors

eAppendix 3. Acknowledgements for EADB Cohorts

eReferences.

This supplemental material has been provided by the authors to give readers additional information about their work.

© 2023 European Alzheimer’s & Dementia Biobank Mendelian Randomization (EADB-MR) Collaboration et al. *JAMA Network Open.*

eMethods.

Selection of instrumental variables

We selected independent single nucleotide polymorphisms (SNPs), i.e., not in linkage disequilibrium with other SNPs for the same risk factor, at genome-wide significance level ($p<5\times10^{-8}$) as instrumental variables for each risk factor. Pairwise linkage disequilibrium was reported in original GWAS, except for BMI which is based on the European 1000 Genome Project reference panel, and we retained the SNP with the smallest p-value. F-statistics for each SNP were computed to quantify instrument strengths, which were between 14.5 and 5567, indicating sufficient strength (>10). Phenotypic variation explained by the selected SNPs were obtained from the original GWAS or calculated for lipid traits[1].

Alzheimer's disease data source

Proxy-AD cases were only identified from the UK Biobank via questionnaire data asking if parents of the participants had AD ("Has/did your father or mother ever suffer from Alzheimer's disease/dementia?"). Participants were categorized into proxy-AD case if the answer was yes, otherwise controls. The last analysis was performed to investigate the validity of using proxy-AD cases for MR analysis, where designation was based on questionnaire data asking if parents had AD.

Additional sensitivity analyses

Furthermore, to differentiate correlated pleiotropic effects (i.e. a genetic variant affects the outcome and exposure through a shared heritable factor) from the causal effect, we also applied Causal Analysis using Summary Effect Estimates (CAUSE) to the main dataset[2]. This method uses the genome-wide summary statistics of both the exposure and outcome to test the performance of the causal model and non-causal model (the latter called the sharing model, where the causal effect is fixed to zero), and to assess whether the sharing model performs at least as well as the causal model. CAUSE has somewhat lower power than particularly the IVW and MR-PRESSO methods and substantially better power than Egger regression[2].

Since the apolipoprotein E (*APOE)* gene, mapped to chromosome 19, is well-acknowledged in the pathogenesis of AD, we repeated the main analyses after stringently excluding genetic variants located on chromosome 19. Furthermore, to account for sample overlap between the exposure and the outcome, we used cross-trait linkage disequilibrium-score regression to approximate the overlap as described previously[3], thereby reducing and correcting for bias in IVW estimation resulting from winner's curse and weak instrument bias. Significant associations between modifiable risk factors and AD obtained by the IVW method were adjusted for potential overlap bias. To evaluate possible reverse causation, we performed MR analyses to assess the effect of genetic predisposition to high AD risk on behavioral risk factors, including educational attainment, smoking, alcohol consumption, and BMI, using the same GWAS depicted above. A total of 83 independent (linkage disequilibrium < 0.001) genome-wide significant variants ($p<5\times10^{-8}$, excluding the region corresponding to *APOE*) from EADB were used as instrumental variables for AD.

© 2023 European Alzheimer's & Dementia Biobank Mendelian Randomization (EADB-MR) Collaboration et al. *JAMA Network Open.*

eAppendix 1. Supplementary Results

In the CAUSE method analysis, the causal models generally trended towards a better fit than the sharing models for both HDL cholesterol, SBP, and DBP; the differences were however not statistically significant (eTable 5 in Supplement 1). This is likely due to the fact that this method uses the entire genome-wide summary statistics of both the exposure and outcome, and thus includes large amounts of insignificant genetic variants, resulting in substantially reduced power compared to the IVW and MR PRESSO methods.

Results after removing SNPs on chromosome 19 remained similar to the main analyses (eFigures 5 to 6 in Supplement 1). After adjusting for sample overlap in the EADB-diagnosed dataset using cross-trait linkage disequilibrium, the corrected estimates did not differ substantially; the ORs were 1.08 (1.02-1.14) for high HDL cholesterol, 0.84 (0.80-0.88) for high educational attainment, 0.95 (0.88-1.01) for smoking initiation, and 0.94 (0.86-1.02) for high BMI, respectively. Furthermore, genetic predisposition to higher odds of AD were not associated with educational attainment, smoking, alcohol consumption, or BMI (eTable 6 in Supplement 1).

eAppendix 2. Supplementary Discussion

First, high SBP may lead to brain vascular abnormalities such as atherosclerotic plaques, contributing to brain ischemia and infarctions. Second, the opposite directions of the SBP and DBP associations may lie in that long-term high SBP is correlated with left ventricular mass and wall thickness[4] which is linked to diastolic dysfunction characterized by lower DBP[5]. Particularly, left ventricular mass increase is associated with cognitive decline and dementia independent of BP[6]. Third, arterial stiffness, characterized by higher SBP and lower DBP, leads to the reduced cerebral blood flow that is a main caveat for cerebral hypoperfusion and associates with neurodegenerative damage. Additionally, high SBP, might be directly involved in AD pathologies, including brain atrophy of abnormal white matter lesion volumes[7] and cerebral amyloid burden[8], whereas low DBP may play a role in cognitive impairment and dementia mediated by tau-pathologies[9].

© 2023 European Alzheimer's & Dementia Biobank Mendelian Randomization (EADB-MR) Collaboration et al. *JAMA Network Open.*

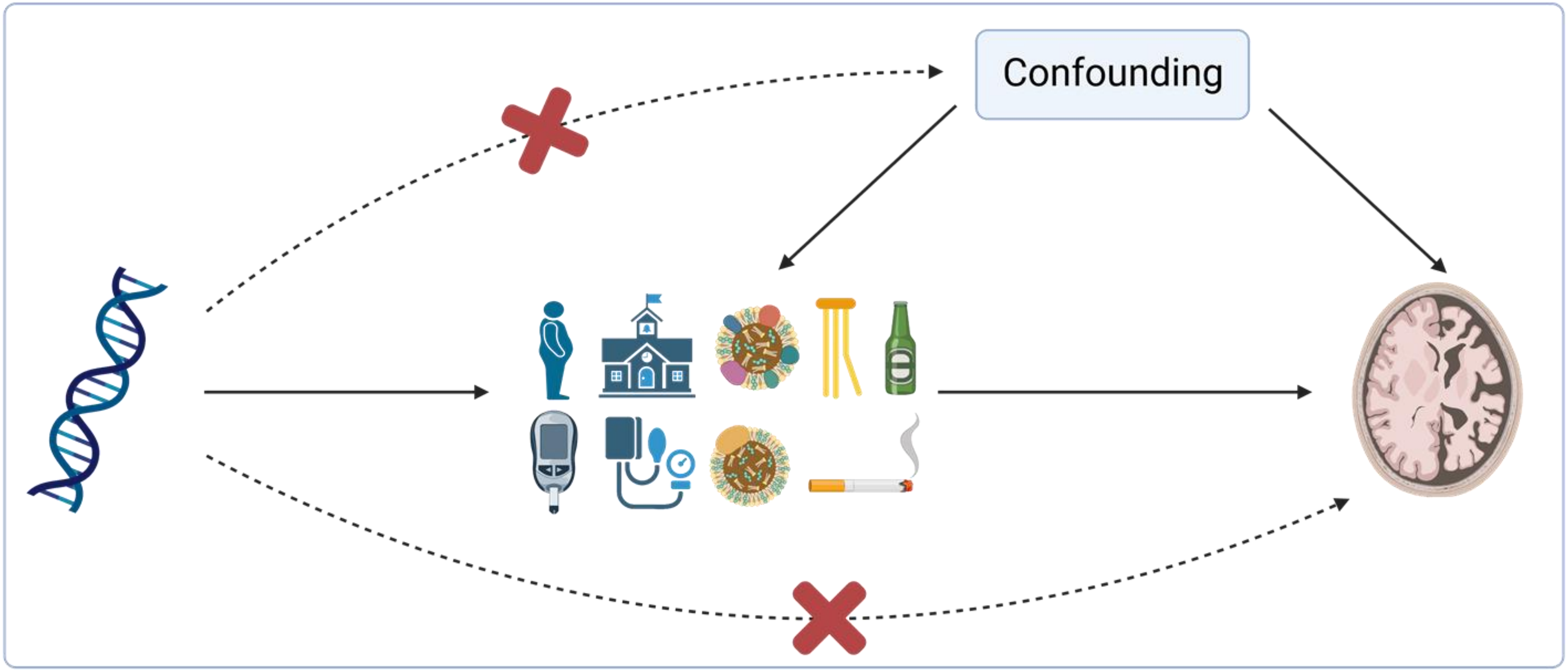

eFigure 1. The Concept of Mendelian Randomization Design

In the Mendelian randomization (MR) design, genetic variants associated with different modifiable risk factors at a genome-wide significant level from the genome-wide association studies were exploited as instrumental variables for these risk factors. Since genetic variants are randomly allocated at conception, the MR strategy is largely free of confounding factors and reverse causation – two common culprits in conventional observational analyses. MR builds on three principal assumptions: the instrumental variables should firstly be associated with different modifiable risk factors; secondly not be associated with confounding factors in the relation between different modifiable risk factors and Alzheimer's disease (AD); thirdly affect AD exclusively via different modifiable risk factors, but not via other pathways. The potential causal effect of different modifiable risk factors on AD could be estimated by dividing gene-AD associations by gene-risk factor associations.

© 2023 European Alzheimer's & Dementia Biobank Mendelian Randomization (EADB-MR) Collaboration et al. *JAMA Network Open.*

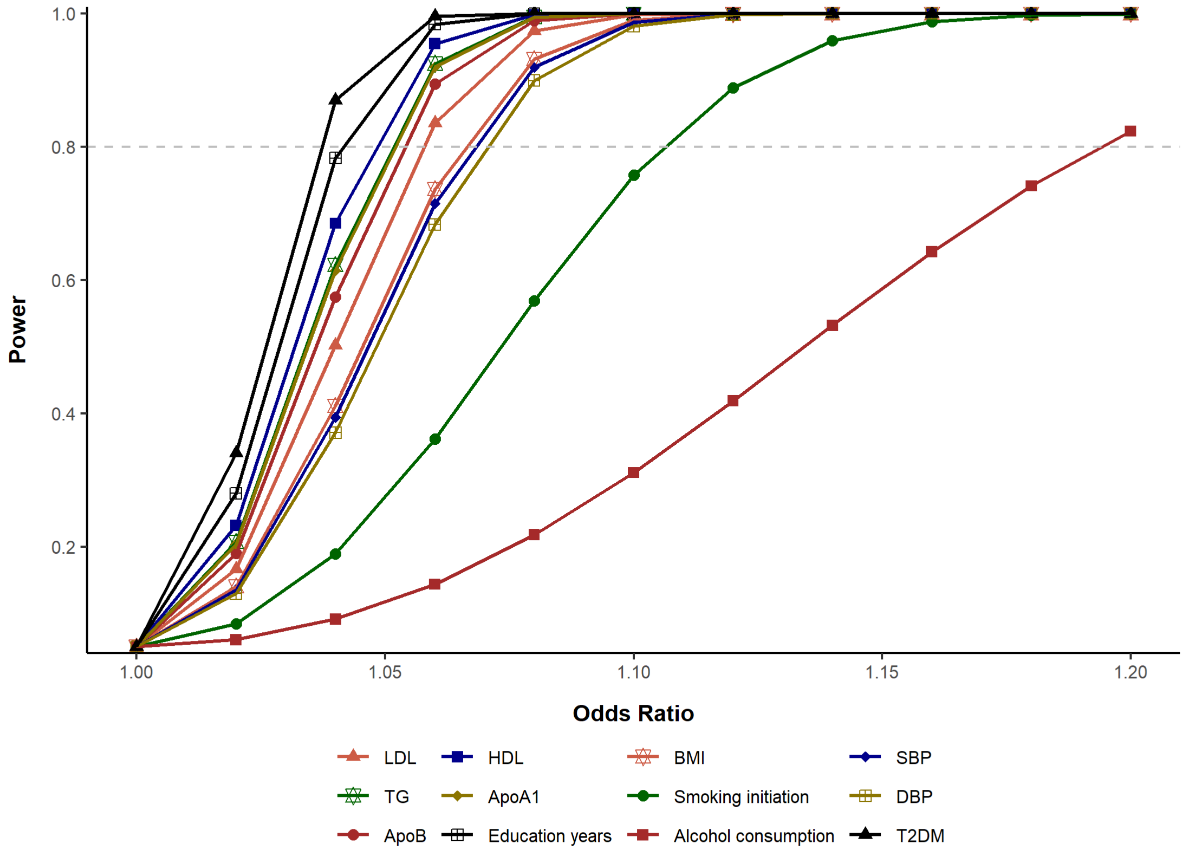

eFigure 2. Statistical Power of Each Exposure in the EADB Consortium

© 2023 European Alzheimer's & Dementia Biobank Mendelian Randomization (EADB-MR) Collaboration et al. *JAMA Network Open.*

Risk factors	No. of SNPs	OR (95% CI)	MR-Egger intercept (p-val)
**LDL (per-SD increase)**			
Inverse variance weighted	166	0.93 (0.86, 1.01)	
Weighted median	166	0.89 (0.81, 0.98)	
MR Egger	166	0.87 (0.75, 1.00)	0.003 (0.2)
MR PRESSO	160	0.93 (0.87, 1.00)	
Multivariable MR (adjusted for HDL and TG)	196	0.93 (0.86, 1.01)	
**ApoB (per-SD increase)**			
Inverse variance weighted	183	0.95 (0.88, 1.02)	
Weighted median	183	0.95 (0.86, 1.04)	
MR Egger	183	0.87 (0.77, 0.99)	0.003 (0.1)
MR PRESSO	181	0.94 (0.88, 1.01)	
Multivariable MR (adjusted for HDL and TG)	240	0.95 (0.88, 1.01)	
**TG (per-SD increase)**			
Inverse variance weighted	340	0.97 (0.92, 1.02)	
Weighted median	340	0.94 (0.88, 1.01)	
MR Egger	340	0.97 (0.89, 1.05)	0 (0.9)
MR PRESSO	335	0.97 (0.93, 1.02)	
Multivariable MR (adjusted for HDL and LDL)	408	1.04 (0.98, 1.11)	
**HDL (per-SD increase)**			
Inverse variance weighted	411	1.10 (1.04, 1.15)	
Weighted median	411	1.12 (1.03, 1.21)	
MR Egger	411	1.03 (0.95, 1.11)	0.002 (0)
MR PRESSO	400	1.13 (1.08, 1.18)	
Multivariable MR (adjusted for LDL and TG)	492	1.08 (1.02, 1.14)	
**ApoA1 (per-SD increase)**			
Inverse variance weighted	337	1.07 (1.00, 1.13)	
Weighted median	337	1.08 (1.00, 1.16)	
MR Egger	337	0.93 (0.85, 1.02)	0.005 (0)
MR PRESSO	324	1.12 (1.06, 1.18)	
Multivariable MR (adjusted for LDL and TG)	416	1.07 (1.02, 1.13)	
**Education (years)**			
Inverse variance weighted	3197	1.06 (1.01, 1.10)	
Weighted median	3197	1.05 (0.99, 1.12)	
MR Egger	3197	1.04 (0.91, 1.19)	0 (0.8)
MR PRESSO	3186	1.06 (1.02, 1.11)	
**Smoking (smoking regularly v.s never smoke)**			
Inverse variance weighted	312	0.88 (0.82, 0.94)	
Weighted median	312	0.89 (0.81, 0.97)	
MR Egger	312	0.75 (0.56, 1.01)	0.003 (0.3)
MR PRESSO	310	0.88 (0.82, 0.94)	
**Alcoholic drinks (per-SD increase per week)**			
Inverse variance weighted	78	0.93 (0.74, 1.15)	
Weighted median	78	1.03 (0.79, 1.33)	
MR Egger	78	0.96 (0.66, 1.39)	-0.001 (0.8)
MR PRESSO	75	0.89 (0.74, 1.07)	
**BMI (per-SD increase)**			
Inverse variance weighted	440	0.89 (0.83, 0.95)	
Weighted median	440	0.86 (0.77, 0.95)	
MR Egger	440	0.87 (0.73, 1.03)	0 (0.6)
MR PRESSO	435	0.89 (0.84, 0.95)	
**SBP (per-10 mmHg increase)**			
Inverse variance weighted	215	0.98 (0.93, 1.03)	
Weighted median	215	0.98 (0.91, 1.05)	
MR Egger	215	0.97 (0.85, 1.11)	0 (0.9)
MR PRESSO	213	0.98 (0.93, 1.03)	
Multivariable MR (adjusted for DBP)	308	1.10 (0.94, 1.30)	
**DBP (per-10 mmHg increase)**			
Inverse variance weighted	268	0.85 (0.78, 0.92)	
Weighted median	268	0.87 (0.77, 0.98)	
MR Egger	268	0.76 (0.62, 0.94)	0.002 (0.3)
MR PRESSO	263	0.83 (0.76, 0.89)	
Multivariable MR (adjusted for SBP)	364	0.73 (0.58, 0.91)	
**T2DM (per unit in log odds)**			
Inverse variance weighted	353	1.00 (0.97, 1.03)	
Weighted median	353	0.99 (0.94, 1.04)	
MR Egger	353	0.99 (0.94, 1.05)	0 (0.8)
MR PRESSO	347	0.99 (0.96, 1.01)	

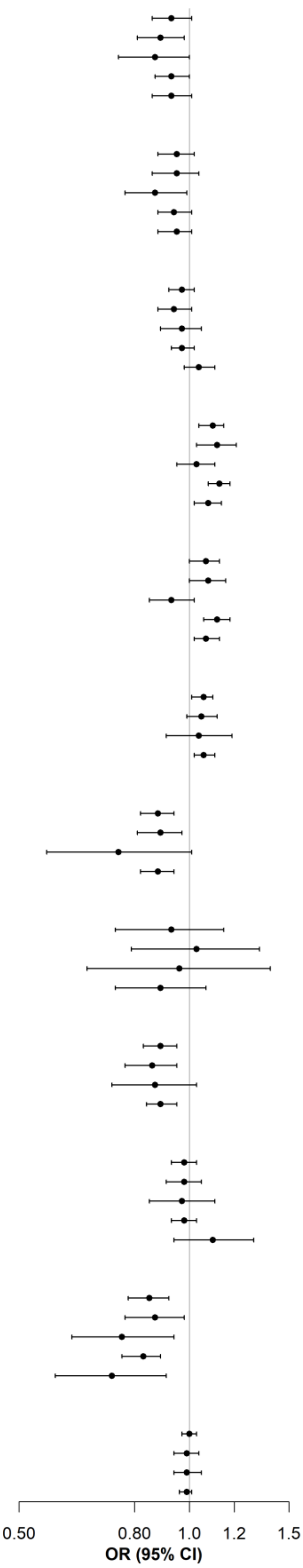

© 2023 European Alzheimer's & Dementia Biobank Mendelian Randomization (EADB-MR) Collaboration et al. *JAMA Network Open.*

eFigure 3. Associations of Genetically Determined Modifiable Risk Factors and AD in the EADB-Proxy Data Set

Multivariable Mendelian randomization was performed for correlated phenotypes only (lipid traits and blood pressure). apoA1: apolipoprotein A1; apoB: apolipoprotein B; BMI: body mass index; CI: confidence interval; EADB: European Alzheimer's & Dementia Biobank; HDL: high-density lipoprotein cholesterol; LDL: low-density lipoprotein cholesterol; OR: odds ratio; SD: standard deviation; SNP: single nucleotide polymorphisms; T2DM: type 2 diabetes mellitus; TG: triglycerides.

© 2023 European Alzheimer's & Dementia Biobank Mendelian Randomization (EADB-MR) Collaboration et al. *JAMA Network Open.*

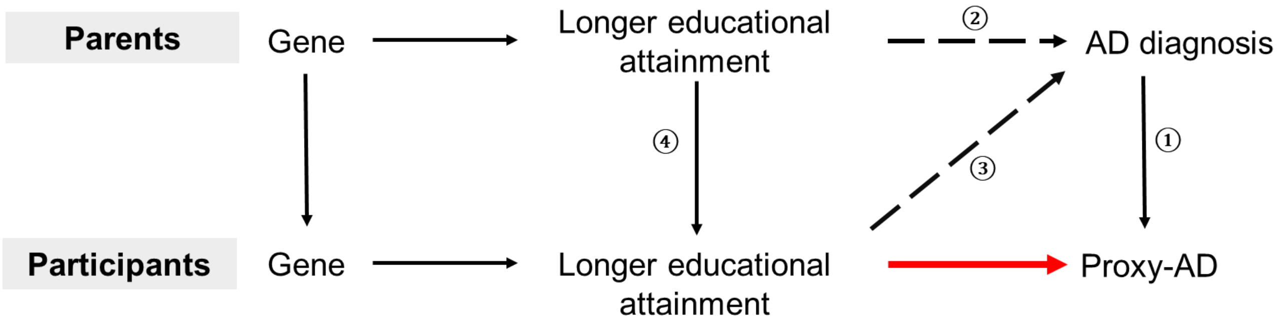

eFigure 4. Directed Acyclic Graph Illustration on the Association Between Educational Attainment and Proxy-AD

Proxy-AD cases in the UK Biobank were identified via questionnaire data asking if parents had AD (arrow①). Despite the fact that longer educational attainment is a protective factor for developing AD, people with longer educational attainment are more prone to get diagnosed compared with those with shorter educational attainment (arrow②). Participants with longer educational attainment may have a tendency to report their parental AD status more precisely (arrow③). Longer educational attainment of the parents, often representing a high social-economic status, is possibly associated with the educational attainment of the participants (arrow④). Therefore, the backdoor path from the participants' educational attainment to AD status (proxy) opens via the parental educational attainment and parents' AD status. In addition, the genetic variants associated with the participants' educational attainment may also be associated with parental educational attainment through the parental genetic variants associated with parental educational attainment. This violates the assumption of Mendelian randomization, and consequently leads to a spurious association between educational attainment of participants and risk of the proxy-AD endpoint. AD: Alzheimer's disease.

© 2023 European Alzheimer's & Dementia Biobank Mendelian Randomization (EADB-MR) Collaboration et al. *JAMA Network Open.*

Risk factors	No. of SNPs	OR (95% CI)	MR-Egger intercept (p-val)
**LDL (per-SD increase)**			
Inverse variance weighted	155	0.99 (0.90, 1.08)	
Weighted median	155	0.92 (0.83, 1.03)	
MR Egger	155	0.93 (0.79, 1.08)	0.002 (0.3)
MR PRESSO	152	0.97 (0.89, 1.06)	
Multivariable MR (adjusted for HDL and TG)	197	0.98 (0.90, 1.07)	
**ApoB (per-SD increase)**			
Inverse variance weighted	171	1.00 (0.93, 1.08)	
Weighted median	171	0.98 (0.86, 1.11)	
MR Egger	171	0.93 (0.81, 1.06)	0.003 (0.2)
MR PRESSO	170	1.01 (0.94, 1.09)	
Multivariable MR (adjusted for HDL and TG)	241	1.00 (0.93, 1.08)	
**TG (per-SD increase)**			
Inverse variance weighted	321	1.02 (0.96, 1.08)	
Weighted median	321	1.01 (0.93, 1.10)	
MR Egger	321	1.04 (0.95, 1.15)	-0.001 (0.5)
MR PRESSO	314	1.02 (0.96, 1.08)	
Multivariable MR (adjusted for HDL and LDL)	409	1.06 (0.98, 1.14)	
**HDL (per-SD increase)**			
Inverse variance weighted	394	1.06 (1.00, 1.13)	
Weighted median	394	1.08 (1.00, 1.18)	
MR Egger	394	1.00 (0.91, 1.09)	0.002 (0.1)
MR PRESSO	386	1.07 (1.01, 1.13)	
Multivariable MR (adjusted for LDL and TG)	493	1.07 (1.01, 1.14)	
**ApoA1 (per-SD increase)**			
Inverse variance weighted	324	1.03 (0.96, 1.10)	
Weighted median	324	1.08 (0.98, 1.19)	
MR Egger	324	0.92 (0.82, 1.02)	0.004 (0)
MR PRESSO	315	1.07 (1.01, 1.14)	
Multivariable MR (adjusted for LDL and TG)	416	1.05 (0.99, 1.11)	
**Education (years)**			
Inverse variance weighted	3149	0.83 (0.79, 0.87)	
Weighted median	3149	0.83 (0.77, 0.89)	
MR Egger	3149	0.79 (0.68, 0.92)	0.001 (0.5)
MR PRESSO	3144	0.84 (0.80, 0.88)	
**Smoking (smoking regularly v.s never smoke)**			
Inverse variance weighted	308	0.93 (0.86, 1.00)	
Weighted median	308	0.96 (0.88, 1.06)	
MR Egger	308	0.70 (0.51, 0.95)	0.006 (0.1)
MR PRESSO	307	0.93 (0.87, 1.00)	
**Alcoholic drinks (per-SD increase per week)**			
Inverse variance weighted	76	0.85 (0.69, 1.05)	
Weighted median	76	1.11 (0.84, 1.48)	
MR Egger	76	1.08 (0.77, 1.52)	-0.004 (0.1)
MR PRESSO	74	0.85 (0.70, 1.02)	
**BMI (per-SD increase)**			
Inverse variance weighted	430	0.94 (0.87, 1.02)	
Weighted median	430	0.84 (0.74, 0.94)	
MR Egger	430	0.91 (0.74, 1.11)	0.001 (0.7)
MR PRESSO	427	0.93 (0.86, 1.00)	
**SBP (per-10 mmHg increase)**			
Inverse variance weighted	210	1.02 (0.96, 1.09)	
Weighted median	210	1.03 (0.95, 1.12)	
MR Egger	210	1.02 (0.87, 1.20)	0 (1)
MR PRESSO	208	1.02 (0.96, 1.08)	
Multivariable MR (adjusted for DBP)	301	1.22 (1.01, 1.47)	
**DBP (per-10 mmHg increase)**			
Inverse variance weighted	259	0.88 (0.80, 0.97)	
Weighted median	259	0.88 (0.77, 1.01)	
MR Egger	259	0.83 (0.65, 1.05)	0.001 (0.6)
MR PRESSO	257	0.88 (0.80, 0.97)	
Multivariable MR (adjusted for SBP)	354	0.74 (0.57, 0.96)	
**T2DM (per unit in log odds)**			
Inverse variance weighted	346	1.01 (0.98, 1.05)	
Weighted median	346	1.02 (0.96, 1.08)	
MR Egger	346	1.00 (0.93, 1.06)	0.001 (0.6)
MR PRESSO	339	1.01 (0.98, 1.04)	

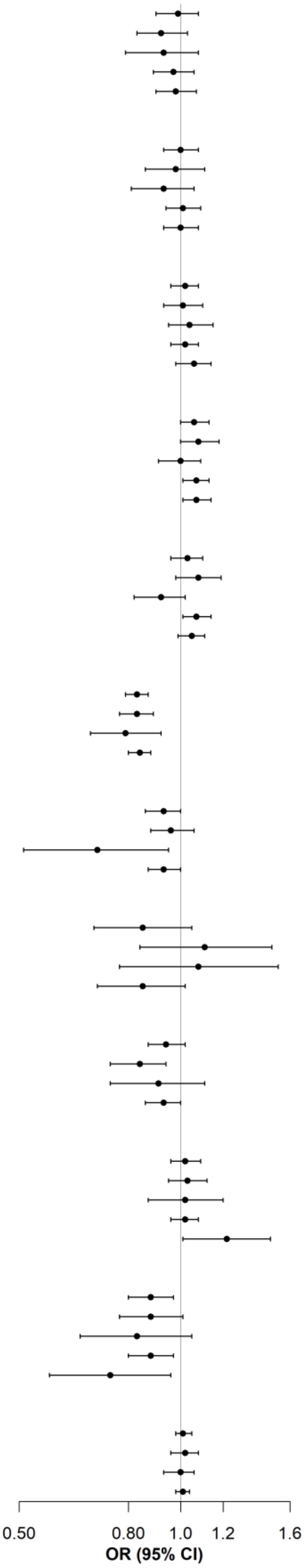

© 2023 European Alzheimer's & Dementia Biobank Mendelian Randomization (EADB-MR) Collaboration et al. *JAMA Network Open.*

eFigure 5. Associations of Genetically Determined Modifiable Risk Factors and AD in the EADB-Diagnosed Data Set Excluding SNVs From Chromosome 19

Multivariable Mendelian randomization was performed for correlated phenotypes only (lipid traits and blood pressure). apoA1: apolipoprotein A1; apoB: apolipoprotein B; BMI: body mass index; CI: confidence interval; EADB: European Alzheimer's & Dementia Biobank; HDL: high-density lipoprotein cholesterol; LDL: low-density lipoprotein cholesterol; OR: odds ratio; SD: standard deviation; SNP: single nucleotide polymorphisms; T2DM: type 2 diabetes mellitus; TG: triglycerides.

© 2023 European Alzheimer's & Dementia Biobank Mendelian Randomization (EADB-MR) Collaboration et al. *JAMA Network Open.*

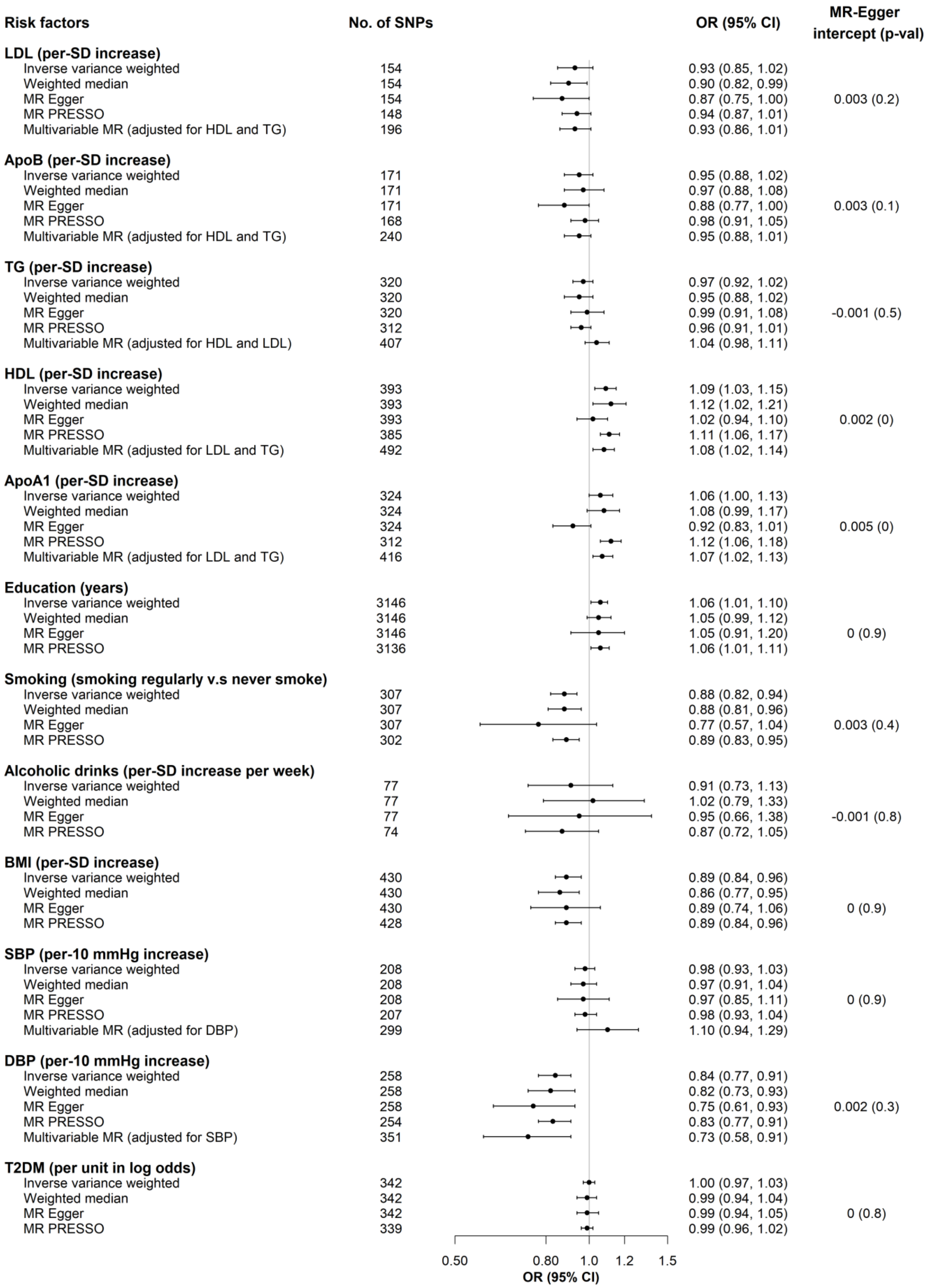

© 2023 European Alzheimer’s & Dementia Biobank Mendelian Randomization (EADB-MR) Collaboration et al. *JAMA Network Open.*

eFigure 6. Associations of Genetically Determined Modifiable Risk Factors and AD in the EADB-Proxy Data Set Excluding SNVs From Chromosome 19

Multivariable Mendelian randomization was performed for correlated phenotypes only (lipid traits and blood pressure). apoA1: apolipoprotein A1; apoB: apolipoprotein B; BMI: body mass index; CI: confidence interval; EADB: European Alzheimer's & Dementia Biobank; HDL: high-density lipoprotein cholesterol; LDL: low-density lipoprotein cholesterol; OR: odds ratio; SD: standard deviation; SNP: single nucleotide polymorphisms; T2DM: type 2 diabetes mellitus; TG: triglycerides.

© 2023 European Alzheimer's & Dementia Biobank Mendelian Randomization (EADB-MR) Collaboration et al. *JAMA Network Open.*

eTable 1. Previous Main Mendelian Randomization Studies on Modifiable Risk Factors and AD Based on Summary Statistics

	**AD consortium**[*]	**Education**	**LDL**[#]	**apoB**	**HDL**[§]	**apoA1**	**TG**	**BMI**	**Alcohol consumption**	**Smoking initiation**	**SBP**	**DBP**	**T2DM**
**Levels**	-	**High**	**Low**	**High**	**High**	**High**	**High**	**High**	**High**	-	**High**	**High**	-
**General modifiable factors**													
Østergaard *et al.*, 2015[10]	IGAP1	×	×		×		×	×		×	↓	×	×
Larsson *et al.*, 2017[11]	IGAP1	↓	×		×		×	×	×	×	×	×	×
Andrews *et al.*, 2020[12]	IGAP2	↓	×		×		×	×	×	×	×	↓[¶]	×
**Specific traits**													
Zhang *et al.*, 2020[13†]	IGAP1	↓	↓		↓		↑	×					
Lord *et al.*, 2021[14†]	IGAP2		×	×	↓	×	×						
Huang *et al.*, 2022[15†]	IGAP2		↓	↑	×	×	↑						
Benn *et al.*, 2017[16]	IGAP1		↓										
Williams *et al.*, 2019[17]	IGAP2 + PGC		↑[‡]										
Nordestgaard *et al.*, 2017[18]	IGAP1							×					
Zhou *et al.*, 2019[19]	IGAP1							×					
Li *et al.*, 2021[20]	ADSP, PGC-ALZ, UKB, IGAP1							↑					
Peloso *et al.*, 2019[21]	IGAP1				×								
Nordestgaard *et al.*, 2022[22]					×								
Kjeldsen *et al.*, 2021[23]	ADSP, PGC-ALZ, UKB, IGAP1				×								
Andrews *et al.*, 2019[24]	IGAP1								×				

© 2023 European Alzheimer's & Dementia Biobank Mendelian Randomization (EADB-MR) Collaboration et al. *JAMA Network Open.*

Sproviero *et al.*, 2021[25]	IGAP1, UKB	↓	↓	
Ou *et al.*, 2021[26]	IGAP2	×	×	
Thomassen *et al.*, 2020[27]	IGAP1			×

AD: Alzheimer’s disease; ADSP: Alzheimer's Disease Sequencing Project; apoA1: apolipoprotein A1; apoB: apolipoprotein B; BMI: body mass index; DBP: diastolic blood pressure; IGAP: International Genomics of Alzheimer’s Project; HDL: high-density lipoprotein cholesterol; LDL: low-density lipoprotein cholesterol; SBP: systolic blood pressure; PGC: Psychiatric Genomics Consortium; T2DM: type 2 diabetes mellitus; TG: triglycerides; UKB: UK Biobank.

[*]IGAP1 is from Lambert *et al.* (2013) and IGAP2 is from Kunkle *et al*. (2019). ×: no association between studied modifiable risk and AD; ↓: per genetically determined one-unit increase of studied modifiable risk factor associated with lower risk of AD; ↑: per genetically determined one-unit increase of studied modifiable risk factor associated with higher risk of AD; blank: no relevant data.

[#]Genetically determined LDL cholesterol concentrations via biochemical measurement or due to *PCSK9* and *HMGCR* variants.

[§]Genetically determined HDL cholesterol concentrations via biochemical measurement or due to *CETP* variants.

[†]Lipid levels in the studies were based on metabolites measured using nuclear magnetic resonance spectroscopy.

[‡]Lipid lowering due to *PCSK9* inhibitor.

[¶]Results from analyses based on polygenic risk scores.

© 2023 European Alzheimer’s & Dementia Biobank Mendelian Randomization (EADB-MR) Collaboration et al. *JAMA Network Open.*

eTable 2. Results From CAUSE Mendelian Randomization

Modifiable risk factor	Model1*	Model2*	Delta ELPD#	se delta ELPD§	z-Score†	p-value‡
HDL cholesterol	Sharing	Causal	-0.13	1.28	-0.10	0.46
Systolic blood pressure	Sharing	Causal	-0.62	1.39	-.044	0.33
Diastolic blood pressure	Sharing	Causal	-0.29	1.34	-0.21	0.42

CAUSE: Causal Analysis Using Summary Effect Estimates; ELPD: Expected Log Pointwise Posterior Density; HDL: high-density lipoprotein cholesterol.
*Model1, model2: the models being compared (sharing or causal).
# Estimated difference in ELPD. If the value is negative, model 2 is a better fit than model 1.
§ Estimated standard error of delta ELPD
† Obtained through (Delta ELPD)/(se delta ELPD).
‡ Obtained by comparing the corresponding z-scores to a normal distribution to test if the difference in model fit is significant.

© 2023 European Alzheimer's & Dementia Biobank Mendelian Randomization (EADB-MR) Collaboration et al. *JAMA Network Open.*

eTable 3. Association of Genetic Predisposition to High Risk of AD and Behavioral Risk Factors

MR method	No. of SNPs	p-value	Estimate	MR Egger intercept (pval)
**Alcoholic drinks per week**				
Inverse variance weighted	72	0.06	1.01 (1.00, 1.02)	
Weighted median	72	0.47	1.00 (0.99, 1.02)	
MR Egger	72	0.31	1.01 (0.99, 1.03)	0 (0.8)
MR PRESSO (outlier-corrected)	71	0.11	1.01 (1.00, 1.01)	
**Body mass index**				
Inverse variance weighted	39	0.92	1.00 (0.98, 1.02)	
Weighted median	39	0.42	0.99 (0.98, 1.01)	
MR Egger	39	0.22	1.04 (0.98, 1.11)	-0.003 (0.2)
MR PRESSO (outlier-corrected)	32	0.77	1.00 (0.98, 1.01)	
**Smoking initiation**				
Inverse variance weighted	74	0.95	1.00 (0.98, 1.02)	
Weighted median	74	0.27	1.01 (0.99, 1.04)	
MR Egger	74	0.96	1.00 (0.96, 1.04)	0 (0.9)
MR PRESSO (outlier-corrected)	73	0.55	1.01 (0.99, 1.02)	
**Education attainment**				
Inverse variance weighted	72	0.28	1.01 (1.00, 1.02)	
Weighted median	72	0.19	1.01 (1.00, 1.02)	
MR Egger	72	0.35	0.99 (0.96, 1.01)	0.001 (0.1)
MR PRESSO (outlier-corrected)	69	0.02	1.01 (1.00, 1.02)	

MR: Mendelian randomization; SNP: single nucleotide polymorphisms.

© 2023 European Alzheimer’s & Dementia Biobank Mendelian Randomization (EADB-MR) Collaboration et al. *JAMA Network Open.*

eAppendix 3. Acknowledgements for EADB Cohorts

EADB-core
The work for this manuscript was further supported by the CoSTREAM project (www.costream.eu) and funding from the European Union's Horizon 2020 research and innovation programme under grant agreement No 667375. This work is also funded by la fondation pour la recherché médicale (FRM) (EQU202003010147) Italian Ministry of Health (Ricerca Corrente); Ministero dell'Istruzione, del l'Università e della Ricerca–MIUR project "Dipartimenti di Eccellenza 2018–2022" to Department of Neuroscience "Rita Levi Montalcini", University of Torino (IR), and AIRAlzh Onlus-ANCC-COOP (SB); Partly supported by "Ministero della Salute", I.R.C.C.S. Research Program, Ricerca Corrente 2018-2020, Linea n. 2 "Meccanismi genetici, predizione e terapie innovative delle malattie complesse" and by the "5 x 1000" voluntary contribution to the Fondazione I.R.C.C.S. Ospedale "Casa Sollievo della Sofferenza"; and RF-2018-12366665, Fondi per la ricercar 2019 (Sandro Sorbi). Copenhagen General Population Study (CGPS): We thank staff and participants of the CGPS for their important contributions. Karolinska Institutet AD cohort: Dr. C.G. and co-authors of the Karolinska Institutet AD cohort report grants from Swedish Research Council (VR) 2015-02926, 2018-02754, 2015-06799, Swedish Alzheimer Foundation, Stockholm County Council ALF and resarch school, Karolinska Institutet StratNeuro, Swedish Demensfonden, and Swedish brain foundation, during the conduct of the study. ADGEN: Academy of Finland (grant number 338182), Sigrid Jusélius Foundation, and the Strategic Neuroscience Funding of the University of Eastern Finland to M. Hiltunen; EADB project in the JPNDCO-FUND program (grant number 301220). CBAS: Supported by the project no. LQ1605 from the National Program of Sustainability II (MEYS CR), Supported by Ministry of Health of the Czech Republic, grant nr. NV19-04-00270 (All rights reserved), Grant Agency of Charles University Grants No. 693018 and 654217; the Ministry of Health, Czech Republic—conceptual development of research organization, University Hospital Motol, Prague, Czech Republic Grant No. 00064203; the Czech Ministry of Health Project AZV Grant No. 16—27611A; and Institutional Support of Excellence 2. LF UK Grant No. 699012. CNRMAJ-Rouen: This study received fundings from the Centre National de Référence Malades Alzheimer Jeunes (CNRMAJ). The Finnish Geriatric Intervention Study for the Prevention of Cognitive Impairment and Disability (FINGER) data collection was supported by grants from the Academy of Finland, La Carita Foundation, Juho Vainio Foundation, Novo Nordisk Foundation, Finnish Social Insurance Institution, Ministry of Education and Culture Research Grants, Yrjö Jahnsson Foundation, Finnish Cultural Foundation South Osthrobothnia Regional Fund, and EVO/State Research Funding grants of University Hospitals of Kuopio, Oulu and Turku, Seinäjoki Central Hospital and Oulu City Hospital, Alzheimer's Research & Prevention Foundation USA, AXA Research Fund, Knut and Alice Wallenberg Foundation Sweden, Center for Innovative Medicine (CIMED) at Karolinska Institutet Sweden, and Stiftelsen Stockholms sjukhem Sweden. FINGER cohort genotyping was funded by EADB project in the JPND CO-FUND (grant number 301220). Research at the Belgian EADB site is funded in part by the Alzheimer Research Foundation (SAO-FRA), The Research Foundation Flanders (FWO), and the University of Antwerp Research Fund. FK is supported by a BOF DOCPRO fellowship of the University of Antwerp Research Fund. SNAC-K is financially supported by the Swedish Ministry of Health and Social Affairs, the participating County Councils and Municipalities, and the Swedish Research Council. BDR Bristol: We would like to thank the South West Dementia Brain Bank (SWDBB) for providing brain tissue for this study. The SWDBB is part of the Brains for Dementia Research programme, jointly funded by Alzheimer's Research UK and Alzheimer's Society and is supported by BRACE (Bristol Research into Alzheimer's and Care of the Elderly) and the Medical Research Council. BDR Manchester: We would like to thank the Manchester Brain Bankfor providing brain tissue for this study. The Manchester Brain Bank is part of the Brains for Dementia Research programme, jointly funded by Alzheimer's Research UK and Alzheimer's Society. BDR KCL: Human post-mortem

© 2023 European Alzheimer's & Dementia Biobank Mendelian Randomization (EADB-MR) Collaboration et al. *JAMA Network Open.*

tissue was provided by the London Neurodegenerative Diseases Brain Bank which receives funding from the UK Medical Research Council and as part of the Brains for Dementia Research programme, jointly funded by Alzheimer's Research UK and the Alzheimer's Society. The CFAS Wales study was funded by the ESRC (RES-060-25-0060) and HEFCW as 'Maintaining function and well-being in later life: a longitudinal cohort study'. We are grateful to the NISCHR Clinical Research Centre for their assistance in tracing participants and in interviewing and in collecting blood samples, and to general practices in the study areas for their cooperation. MRC: We thank all individuals who participated in this study. Cardiff University was supported by the Alzheimer's Society (AS; grant RF014/164) and the Medical Research Council (MRC; grants G0801418/1, MR/K013041/1, MR/L023784/1) (R.S. is an AS Research Fellow). Cardiff University was also supported by the European Joint Programme for Neurodegenerative Disease (JPND; grant MR/L501517/1), Alzheimer's Research UK (ARUK; grant ARUK-PG2014-1), the Welsh Assembly Government (grant SGR544: CADR), Brain's for dementia Research and a donation from the Moondance Charitable Foundation. Cardiff University acknowledges the support of the UK Dementia Research Institute, of which J.W. is an associate director. Cambridge University acknowledges support from the MRC. Patient recruitment for the MRC Prion Unit/UCL Department of Neurodegenerative Disease collection was supported by the UCLH/UCL Biomedical Centre and NIHR Queen Square Dementia Biomedical Research Unit. The University of Southampton acknowledges support from the AS. King's College London was supported by the NIHR Biomedical Research Centre for Mental Health and the Biomedical Research Unit for Dementia at the South London and Maudsley NHS Foundation Trust and by King's College London and the MRC. ARUK and the Big Lottery Fund provided support to Nottingham University. A.Ram. : Part of the work was funded by the JPND EADB grant (German Federal Ministry of Education and Research (BMBF) grant: 01ED1619A). A. Ram. is also supported by the German Research Foundation (DFG) grants Nr: RA 1971/6-1, RA1971/7-1, and RA 1971/8-1. German Study on Ageing, Cognition and Dementia in Primary Care Patients (AgeCoDe): This study/publication is part of the German Research Network on Dementia (KND), the German Research Network on Degenerative Dementia (KNDD; German Study on Ageing, Cognition and Dementia in Primary Care Patients; AgeCoDe), and the Health Service Research Initiative (Study on Needs, health service use, costs and health-related quality of life in a large sample of oldestold primary care patients (85+; AgeQualiDe)) and was funded by the German Federal Ministry of Education and Research (grants KND: 01GI0102, 01GI0420, 01GI0422, 01GI0423, 01GI0429, 01GI0431, 01GI0433, 01GI0434; grants KNDD: 01GI0710, 01GI0711, 01GI0712, 01GI0713, 01GI0714, 01GI0715, 01GI0716; grants Health Service Research Initiative: 01GY1322A, 01GY1322B, 01GY1322C, 01GY1322D, 01GY1322E, 01GY1322F, 01GY1322G). VITA study: The support of the Ludwig Boltzmann Society and the AFI Germany have supported the VITA study. The former VITA study group should be acknowledged: W. Danielczyk, G. Gatterer, K Jellinger, S Jugwirth, KH Tragl, S Zehetmayer. Vogel Study: This work was financed by a research grant of the ''Vogelstiftung Dr. Eckernkamp''. HELIAD study: This study was supported by the grants: IIRG-09-133014 from the Alzheimer's Association, 18910276/8/9/2011 from the ESPA-EU program Excellence Grant (ARISTEIA) and the ΔΥ2β/οικ.51657/14.4.2009 of the Ministry for Health and Social Solidarity (Greece). Biobank Department of Psychiatry, UMG: Prof. Jens Wiltfang is supported by an Ilídio Pinho professorship and iBiMED (UID/BIM/04501/2013), and FCT project PTDC/DTP_PIC/5587/2014 at the University of Aveiro, Portugal. Lausanne study: This work was supported by grants from the Swiss National Research Foundation (SNF 320030_141179). PAGES study: Harald Hampel is an employee of Eisai Inc. During part of this work he was supported by the AXA Research Fund, the "Fondation partenariale Sorbonne Université" and the "Fondation pour la Recherche sur Alzheimer", Paris, France. Mannheim, Germany Biobank: Department of geriatric Psychiatry, Central Institute for Mental Health, Mannheim, University of Heidelberg, Germany. Genotyping for the Swedish Twin Studies of Aging was supported by NIH/NIA grant R01 AG037985. Genotyping in TwinGene was supported by

© 2023 European Alzheimer's & Dementia Biobank Mendelian Randomization (EADB-MR) Collaboration et al. *JAMA Network Open.*

NIH/NIDDK U01 DK066134. WvdF is recipient of Joint Programming for Neurodegenerative Diseases (JPND) grants PERADES (ANR-13-JPRF-0001) and EADB (733051061). Gothenburg Birth Cohort (GBC) Studies: We would like to thank UCL Genomics for performing the genotyping analyses. The studies were supported by The Stena Foundation, The Swedish Research Council (2015-02830, 2013-8717), The Swedish Research Council for Health, Working Life and Wellfare (2013-1202, 2005-0762, 2008-1210, 2013-2300, 2013- 2496, 2013-0475), The Brain Foundation, Sahlgrenska University Hospital (ALF), The Alzheimer's Association (IIRG-03-6168), The Alzheimer's Association Zenith Award (ZEN-01-3151), Eivind och Elsa K:son Sylvans Stiftelse, The Swedish Alzheimer Foundation. Clinical AD, Sweden: We would like to thank UCL Genomics for performing the genotyping analyses. Barcelona Brain Biobank: Brain Donors of the Neurological Tissue Bank of the Biobanc-Hospital Clinic-IDIBAPS and their families for their generosity. Hospital Clínic de Barcelona Spanish Ministry of Economy and Competitiveness-Instituto de Salud Carlos III and Fondo Europeo de Desarrollo Regional (FEDER), Unión Europea, "Una manera de hacer Europa" grants (PI16/0235 to Dr. R. Sánchez-Valle and PI17/00670 to Dr. A.Antonelli). AA is funded by Departament de Salut de la Generalitat de Catalunya, PERIS 2016-2020 (SLT002/16/00329). Work at JP-T laboratory was possible thanks to funding from Ciberned and generous gifts from Consuelo Cervera Yuste and Juan Manuel Moreno Cervera. This work was supported by InnoMed (Innovative Medicines in Europe), an integrated project funded by the European Union of the Sixth Framework program priority (FP6-2004- LIFESCIHEALTH-5). Oviedo: This work was partly supported by Grant from Fondo de Investigaciones Sanitarias-Fondos FEDER EuropeanUnion to V.A. PI15/00878. Project MinE: The ProjectMinE study was supported by the ALS Foundation Netherlands and the MND association (UK) (Project MinE, www.projectmine.com). The SPIN cohort: We are indebted to patients and their families for their participation in the "Sant Pau Initiative on Neurodegeneration cohort", at the Sant Pau Hospital (Barcelona). This is a multimodal research cohort for biomarker discovery and validation that is partially funded by Generalitat de Catalunya (2017 SGR 547 to JC), as well as from the Institute of Health Carlos III-Subdirección General de Evaluación and the Fondo Europeo de Desarrollo Regional (FEDER- "Una manera de Hacer Europa") (grants PI11/02526, PI14/01126, and PI17/01019 to JF; PI17/01895 to AL), and the Centro de Investigación Biomédica en Red Enfermedades Neurodegenerativas programme (Program 1, Alzheimer Disease to AL). We would also like to thank the Fundació Bancària Obra Social La Caixa (DABNI project) to JF and AL; and Fundación BBVA (to AL), for their support in funding this follow-up study. Adolfo López de Munain is supported by Fundación Salud 2000 (PI2013156), CIBERNED and Diputación Foral de Gipuzkoa (Exp.114/17). P.S.J. is supported by CIBERNED and Carlos III Institute of Health, Spain (PI08/0139, PI12/02288, and PI16/01652, PI20/01011), jointly funded by Fondo Europeo de Desarrollo Regional (FEDER), Unión Europea, "Una manera de hacer Europa". We thank Biobanco Valdecilla for their support. Amsterdam dementia Cohort (ADC): Research of the Alzheimer center Amsterdam is part of the neurodegeneration research program of Amsterdam Neuroscience. The AlzheimerCenter Amsterdam is supported by Stichting Alzheimer Nederland and Stichting VUmc fonds. The clinical database structure was developed with funding from Stichting Dioraphte. Genotyping of the Dutch case-control samples was performed in the context of EADB (European Alzheimer&Dementia biobank) funded by the JPco-fuND FP-829-029 (ZonMW project number #733051061). This research is performed by using data from the Parelsnoer Institute an initiative of the Dutch Federation of University Medical Centres (www.parelsnoer.org). 100-Plus study: We are grateful for the collaborative efforts of all participating centenarians and their family members and/or relations. We thank the Netherlands Brain Bank for supplying DNA for genotyping. This work was supported by Stichting AlzheimerNederland (WE09.2014-03), Stichting Diorapthe, Horstingstuit foundation, Memorabel (ZonMW project number #733050814, #733050512) and Stichting VUmcFonds. Additional support for EADB cohorts: WF, SL, HH are recipients of ABOARD, a public-private partnership receiving funding from ZonMW (#73305095007) and Health~Holland,

© 2023 European Alzheimer's & Dementia Biobank Mendelian Randomization (EADB-MR) Collaboration et al. *JAMA Network Open.*

Topsector Life Sciences & Health (PPP-allowance; #LSHM20106). The DELCODE study was funded by the German Center for Neurodegenerative Diseases (Deutsches Zentrum für Neurodegenerative Erkrankungen (DZNE)), reference number BN012.

Additional EADB cohorts

ADGC. The National Institutes of Health, National Institute on Aging (NIH-NIA) supported this work through the following grants: ADGC, U01 AG032984, RC2 AG036528; Samples from the National Cell Repository for Alzheimer’s Disease (NCRAD), which receives government support under a cooperative agreement grant (U24 AG21886) awarded by the National Institute on Aging (NIA), were used in this study. We thank contributors who collected samples used in this study, as well as patients and their families, whose help and participation made this work possible; Data for this study were prepared, archived, and distributed by the National Institute on Aging Alzheimer’s Disease Data Storage Site (NIAGADS) at the University of Pennsylvania (U24-AG041689-01); NACC, U01 AG016976; NIA LOAD (Columbia University), U24 AG026395, U24 AG026390, R01AG041797; Banner Sun Health Research Institute P30 AG019610; Boston University, P30 AG013846, U01 AG10483, R01 CA129769, R01 MH080295, R01 AG017173, R01 AG025259, R01 AG048927, R01AG33193, R01 AG009029; Columbia University, P50 AG008702, R37 AG015473, R01 AG037212, R01 AG028786; Duke University, P30 AG028377, AG05128; Einstein Aging Study NIA grant at Albert Einstein College of Medicine, P01 AG03949. Emory University, AG025688; Group Health Research Institute, UO1 AG006781, UO1 HG004610, UO1 HG006375, U01 HG008657; Indiana University, P30 AG10133, R01 AG009956, RC2 AG036650; Johns Hopkins University, P50 AG005146, R01 AG020688; Massachusetts General Hospital, P50 AG005134; Mayo Clinic, P50 AG016574, R01 AG032990, KL2 RR024151; Mount Sinai School of Medicine, P50 AG005138, P01 AG002219; New York University, P30 AG08051, UL1 RR029893, 5R01AG012101, 5R01AG022374, 5R01AG013616, 1RC2AG036502, 1R01AG035137; North Carolina A&T University, P20 MD000546, R01 AG28786-01A1; Northwestern University, P30 AG013854; Oregon Health & Science University, P30 AG008017, R01 AG026916; Rush University, P30 AG010161, R01 AG019085, R01 AG15819, R01 AG17917, R01 AG030146, R01 AG01101, RC2 AG036650, R01 AG22018; TGen, R01 NS059873; University of Alabama at Birmingham, P50 AG016582; University of Arizona, R01 AG031581; University of California, Davis, P30 AG010129; University of California, Irvine, P50 AG016573; University of California, Los Angeles, P50 AG016570; University of California, San Diego, P50 AG005131; University of California, San Francisco, P50 AG023501, P01 AG019724; University of Kentucky, P30 AG028383, AG05144; University of Michigan, P30 AG053760 and AG063760; University of Pennsylvania, P30 AG010124; University of Pittsburgh, P50 AG005133, AG030653, AG041718, AG07562, AG02365; University of Southern California, P50 AG005142; University of Texas Southwestern, P30 AG012300; University of Miami, R01 AG027944, AG010491, AG027944, AG021547, AG019757; University of Washington, P50 AG005136, R01 AG042437; University of Wisconsin, P50 AG033514; Vanderbilt University, R01 AG019085; and Washington University, P50 AG005681, P01 AG03991, P01 AG026276. HP was supported by AG025711. ER was supported by CCNA. The Kathleen Price Bryan Brain Bank at Duke University Medical Center is funded by NINDS grant # NS39764, NIMH MH60451 and by Glaxo Smith Kline. Support was also from the Alzheimer’s Association (LAF, IIRG-08-89720; MP-V, IIRG-05- 14147), the US Department of Veterans Affairs Administration, Office of Research and Development, Biomedical Laboratory Research Program, and BrightFocus Foundation (MP-V, A2111048). P.S.G.-H. is supported by Wellcome Trust, Howard Hughes Medical Institute, and the Canadian Institute of Health Research. Genotyping of the TGEN2 cohort was supported by Kronos Science. The TGen series was also funded by NIA grant AG041232 to AJM and MJH, The Banner Alzheimer’s Foundation, The Johnnie B. Byrd Sr. Alzheimer’s Institute, the Medical Research Council, and the

© 2023 European Alzheimer’s & Dementia Biobank Mendelian Randomization (EADB-MR) Collaboration et al. *JAMA Network Open.*

state of Arizona and also includes samples from the following sites: Newcastle Brain Tissue Resource (funding via the Medical Research Council, local NHS trusts and Newcastle University), MRC London Brain Bank for Neurodegenerative Diseases (funding via the Medical Research Council), South West Dementia Brain Bank (funding via numerous sources including the Higher Education Funding Council for England (HEFCE), Alzheimer's Research Trust (ART), BRACE as well as North Bristol NHS Trust Research and Innovation department and DeNDRoN), The Netherlands Brain Bank (funding via numerous sources including Stichting MS Research, Brain Net Europe, Hersenstichting Nederland Breinbrekend Werk, International Parkinson Fonds, Internationale Stiching Alzheimer Onderzoek), Institut de Neuropatologia, Servei Anatomia Patologica, Universitat de Barcelona. ADNI data collection and sharing was funded by the National Institutes of Health Grant U01 AG024904 and Department of Defense award number W81XWH-12-2-0012. ADNI is funded by the National Institute on Aging, the National Institute of Biomedical Imaging and Bioengineering, and through generous contributions from the following: AbbVie, Alzheimer's Association; Alzheimer's Drug Discovery Foundation; Araclon Biotech; BioClinica, Inc.; Biogen; Bristol-Myers Squibb Company; CereSpir, Inc.; Eisai Inc.; Elan Pharmaceuticals, Inc.; Eli Lilly and Company; EuroImmun; F. Hoffmann-La Roche Ltd and its affiliated company Genentech, Inc.; Fujirebio; GE Healthcare; IXICO Ltd.; Janssen Alzheimer Immunotherapy Research & Development, LLC.; Johnson & Johnson Pharmaceutical Research & Development LLC.; Lumosity; Lundbeck; Merck & Co., Inc.; Meso Scale Diagnostics, LLC.; NeuroRx Research; Neurotrack Technologies; Novartis Pharmaceuticals Corporation; Pfizer Inc.; Piramal Imaging; Servier; Takeda Pharmaceutical Company; and Transition Therapeutics. The Canadian Institutes of Health Research is providing funds to support ADNI clinical sites in Canada. Private sector contributions are facilitated by the Foundation for the National Institutes of Health (www.fnih.org). The grantee organization is the Northern California Institute for Research and Education, and the study is coordinated by the Alzheimer's Disease Cooperative Study at the University of California, San Diego. ADNI data are disseminated by the Laboratory for Neuro Imaging at the University of Southern California. We thank Drs. D. Stephen Snyder and Marilyn Miller from NIA who are *ex-officio* ADGC members. FTLD-TDP GWAS: National Institute on Aging (AG101024, AG066597 and AG017586)

Bonn study. This group would like to thank Dr. Heike Koelsch for her scientific support. The Bonn group was funded by the German Federal Ministry of Education and Research (BMBF): Competence Network Dementia (CND) grant number 01GI0102, 01GI0711, 01GI042

CCHS. Participants and staff of the Copenhagen City Heart Study (CCHS) are thanked for their important contributions. The CCHS was supported by the Danish Heart Foundation, the Danish Lung Association, the Velux Foundation, the Research Council of the Danish Medical Association, and the Work-Environment Foundation.

CHARGE. Cardiovascular Health Study (CHS). This CHS research was supported by NHLBI contracts HHSN268201200036C, HHSN268200800007C, HHSN268201800001C, N01HC55222, N01HC85079, N01HC85080, N01HC85081, N01HC85082, N01HC85083, N01HC85086, 75N92021D00006; and NHLBI grants U01HL080295, U01HL130114, R01HL087652, R01HL105756, R01HL103612, R01HL120393 and 75N92021D00006 with additional contribution from the National Institute of Neurological Disorders and Stroke (NINDS). Additional support was provided through R01AG023629, R01AG033193, R01AG15928, R01AG20098, and U01AG049505 from the National Institute on Aging (NIA). A full list of principal CHS investigators and institutions can be found at CHS-NHLBI.org. The provision of genotyping data was supported in part by the National Center for Advancing Translational Sciences, CTSI grant UL1TR001881, and the National Institute of Diabetes and Digestive and Kidney Disease Diabetes Research Center (DRC) grant

© 2023 European Alzheimer's & Dementia Biobank Mendelian Randomization (EADB-MR) Collaboration et al. *JAMA Network Open.*

DK063491 to the Southern California Diabetes Endocrinology Research Center. Framingham Heart Study. This work was supported by the National Heart, Lung, and Blood Institute's Framingham Heart Study (contracts N01-HC-25195 and HHSN268201500001I). This study was also supported by grants from the National Institute on Aging: R01AG033193, U01AG049505, U01AG52409, R01AG054076, RF1AG0059421 (S. Seshadri). S. Seshadri and A.L.D. were also supported by additional grants from the National Institute on Aging (R01AG049607, R01AG033040, RF1AG0061872, U01AG058589) and the National Institute of Neurological Disorders and Stroke (R01-NS017950, NS100605). The content is solely the responsibility of the authors and does not necessarily represent the official views of the US National Institutes of Health.

DemGene. The project has received funding from The Research Council of Norway (RCN) Grant Nos. 213837, 223273, 225989, 248778, and 251134 and EU JPND Program RCN Grant Nos. 237250, 311993, the South-East Norway Health Authority Grant No. 2013-123, the Norwegian Health Association, and KG Jebsen Foundation. The RCN FRIPRO Mobility grant scheme (FRICON) is co-funded by the European Union’s Seventh Framework Programme for research, technological development and demonstration under Marie Curie grant agreement No 608695. European Community’s grant PIAPP-GA-2011-286213 PsychDPC.

EADI. This work has been developed and supported by the LABEX (laboratory of excellence program investment for the future) DISTALZ grant (Development of Innovative Strategies for a Transdisciplinary approach to ALZheimer’s disease) including funding from MEL (Metropole européenne de Lille), ERDF (European Regional Development Fund) and Conseil Régional Nord Pas de Calais. This work was supported by INSERM, the National Foundation for Alzheimer’s disease and related disorders, the Institut Pasteur de Lille and the Centre National de Recherche en Génomique Humaine, CEA, the JPND PERADES, the Laboratory of Excellence GENMED (Medical Genomics) grant no. ANR-10-LABX-0013 managed by the National Research Agency (ANR) part of the Investment for the Future program, and the FP7 AgedBrainSysBio. The Three-City Study was performed as part of collaboration between the Institut National de la Santé et de la Recherche Médicale (Inserm), the Victor Segalen Bordeaux II University and Sanofi-Synthélabo. The Fondation pour la Recherche Médicale funded the preparation and initiation of the study. The 3C Study was also funded by the Caisse Nationale Maladie des Travailleurs Salariés, Direction Générale de la Santé, MGEN, Institut de la Longévité, Agence Française de Sécurité Sanitaire des Produits de Santé, the Aquitaine and Bourgogne Regional Councils, Agence Nationale de la Recherche, ANR supported the COGINUT and COVADIS projects. Fondation de France and the joint French Ministry of Research/INSERM “Cohortes et collections de données biologiques” programme. Lille Génopôle received an unconditional grant from Eisai. The Three-city biological bank was developed and maintained by the laboratory for genomic analysis LAG-BRC - Institut Pasteur de Lille.

FinnGen. The FinnGen project is funded by two grants from Business Finland (HUS 4685/31/2016 and UH 4386/31/2016) and the following industry partners: AbbVie Inc., AstraZeneca UK Ltd, Biogen MA Inc., Celgene Corporation, Celgene International II Sàrl, Genentech Inc., Merck Sharp & Dohme Corp, Pfizer Inc., GlaxoSmithKline Intellectual Property Development Ltd., Sanofi US Services Inc., Maze Therapeutics Inc., Janssen Biotech Inc, and Novartis AG. Following biobanks are acknowledged for the project samples: Auria Biobank (www.auria.fi/biopankki), THL Biobank (www.thl.fi/biobank), Helsinki Biobank (www.helsinginbiopankki.fi), Biobank Borealis of Northern Finland (https://www.ppshp.fi/Tutkimus-ja-opetus/Biopankki/Pages/Biobank-Borealis-briefly-in-English.aspx), Finnish Clinical Biobank Tampere (www.tays.fi/en-US/Research_and_development/Finnish_Clinical_Biobank_Tampere), Biobank of Eastern

© 2023 European Alzheimer’s & Dementia Biobank Mendelian Randomization (EADB-MR) Collaboration et al. *JAMA Network Open.*

Finland (www.ita-suomenbiopankki.fi/en), Central Finland Biobank (www.ksshp.fi/fi-FI/Potilaalle/Biopankki), Finnish Red Cross Blood Service Biobank (www.veripalvelu.fi/verenluovutus/biopankkitoiminta) and Terveystalo Biobank (www.terveystalo.com/fi/Yritystietoa/Terveystalo-Biopankki/Biopankki/). All Finnish Biobanks are members of BBMRI.fi infrastructure (www.bbmri.fi).

GERAD/PERADES. We thank all individuals who participated in this study. Cardiff University was supported by the Wellcome Trust, Alzheimer's Society (AS; grant RF014/164), the Medical Research Council (MRC; grants G0801418/1, MR/K013041/1, MR/L023784/1), the European Joint Programme for Neurodegenerative Disease (JPND, grant MR/L501517/1), Alzheimer's Research UK (ARUK, grant ARUK-PG2014-1), Welsh Assembly Government (grant SGR544:CADR), a donation from the Moondance Charitable Foundation, UK Dementia's Platform (DPUK, reference MR/L023784/1), and the UK Dementia Research Institute at Cardiff. Cambridge University acknowledges support from the MRC. ARUK supported sample collections at the Kings College London, the South West Dementia Bank, Universities of Cambridge, Nottingham, Manchester and Belfast. King's College London was supported by the NIHR Biomedical Research Centre for Mental Health and Biomedical Research Unit for Dementia at the South London and Maudsley NHS Foundation Trust and Kings College London and the MRC. Alzheimer's Research UK (ARUK) and the Big Lottery Fund provided support to Nottingham University. Ulster Garden Villages, AS, ARUK, American Federation for Aging Research, NI R&D Office and the Royal College of Physicians/Dunhill Medical Trust provided support for Queen's University, Belfast. The University of Southampton acknowledges support from the AS. The MRC and Mercer's Institute for Research on Ageing supported the Trinity College group. DCR is a Wellcome Trust Principal Research fellow. The South West Dementia Brain Bank acknowledges support from Bristol Research into Alzheimer's and Care of the Elderly. The Charles Wolfson Charitable Trust supported the OPTIMA group. Washington University was funded by NIH grants, Barnes Jewish Foundation and the Charles and Joanne Knight Alzheimer's Research Initiative. Patient recruitment for the MRC Prion Unit/UCL Department of Neurodegenerative Disease collection was supported by the UCLH/UCL Biomedical Research Centre and their work was supported by the NIHR Queen Square Dementia BRU, the Alzheimer's Research UK and the Alzheimer's Society. LASER-AD was funded by Lundbeck SA. The AgeCoDe study group was supported by the German Federal Ministry for Education and Research grants 01 GI 0710, 01 GI 0712, 01 GI 0713, 01 GI 0714, 01 GI 0715, 01 GI 0716, 01 GI 0717. Genotyping of the Bonn case-control sample was funded by the German centre for Neurodegenerative Diseases (DZNE), Germany. The GERAD Consortium also used samples ascertained by the NIMH AD Genetics Initiative. HH was supported by a grant of the Katharina-Hardt-Foundation, Bad Homburg vor der Höhe, Germany. The KORA F4 studies were financed by Helmholtz Zentrum München; German Research Center for Environmental Health; BMBF; German National Genome Research Network and the Munich Center of Health Sciences. The Heinz Nixdorf Recall cohort was funded by the Heinz Nixdorf Foundation and BMBF. We acknowledge use of genotype data from the 1958 Birth Cohort collection and National Blood Service, funded by the MRC and the Wellcome Trust which was genotyped by the Wellcome Trust Case Control Consortium and the Type-1 Diabetes Genetics Consortium, sponsored by the National Institute of Diabetes and Digestive and Kidney Diseases, National Institute of Allergy and Infectious Diseases, National Human Genome Research Institute, National Institute of Child Health and Human Development and Juvenile Diabetes Research Foundation International. The project is also supported through the following funding organisations under the aegis of JPND - www.jpnd.eu (United Kingdom, Medical Research Council (MR/L501529/1; MR/R024804/1) and Economic and Social Research Council (ES/L008238/1)) and through the Motor Neurone Disease Association. This study represents independent research part funded by the National Institute for Health Research (NIHR) Biomedical Research Centre at South London and Maudsley NHS Foundation Trust and

© 2023 European Alzheimer's & Dementia Biobank Mendelian Randomization (EADB-MR) Collaboration et al. *JAMA Network Open.*

King’s College London. Prof Jens Wiltfang is supported by an Ilídio Pinho professorship and iBiMED (UID/BIM/04501/2013), at the University of Aveiro, Portugal.

Gra@ce. The Genome Research @ Ace Alzheimer Center Barcelona project (GR@ACE) is supported by Grifols SA, Fundación bancaria ‘La Caixa’, Ace Alzheimer Center Barcelona and CIBERNED. We are indebted to Trinitat Port-Carbó legacy and her family for their support of Ace Alzheimer Center Barcelona research programs. Ace Alzheimer Center Barcelona is one of the participating centers of the Dementia Genetics Spanish Consortium (DEGESCO). A.R. and M.B. receive support from the European Union/EFPIA Innovative Medicines Initiative Joint undertaking ADAPTED and MOPEAD projects (grant numbers 115975 and 115985, respectively). M.B. and A.R. are also supported by national grants PI13/02434, PI16/01861, PI17/01474, PI19/01240 and PI19/01301. Acción Estratégica en Salud is integrated into the Spanish National R + D + I Plan and funded by ISCIII (Instituto de Salud Carlos III)–Subdirección General de Evaluación and the Fondo Europeo de Desarrollo Regional (FEDER–‘Una manera de hacer Europa’). The position held by I.dR. is supported by national grant from the Instituto de Salud Carlos III FI20/00215. Some control samples and data from patients included in this study were provided in part by the National DNA Bank Carlos III (www.bancoadn.org, University of Salamanca, Spain) and Hospital Universitario Virgen de Valme (Sevilla, Spain); they were processed following standard operating procedures with the appropriate approval of the Ethical and Scientific Committee.

Rotterdam study. Rotterdam (RS). This study was funded by the Netherlands Organisation for Health Research and Development (ZonMW) as part of the Joint Programming for Neurological Disease (JPND) as part of the PERADES Program (Defining Genetic Polygenic, and Environmental Risk for Alzheimer’s disease using multiple powerful cohorts, focused Epigenetics and Stem cell metabolomics), Project number 733051021. This work was funded also by the European Union Innovative Medicine Initiative (IMI) programme under grant agreement No. 115975 as part of the Alzheimer’s Disease Apolipoprotein Pathology for Treatment Elucidation and Development (ADAPTED, https://www.imi-adapted.eu) and the European Union’s Horizon 2020 research and innovation programme as part of the Common mechanisms and pathways in Stroke and Alzheimer’s disease CoSTREAM project (www.costream.eu, grant agreement No. 667375). The current study is supported by the Deltaplan Dementie and Memorabel supported by ZonMW (Project number 733050814) and Alzheimer Nederland. The Rotterdam Study is funded by Erasmus Medical Center and Erasmus University, Rotterdam, Netherlands Organization for the Health Research and Development (ZonMw), the Research Institute for Diseases in the Elderly (RIDE), the Ministry of Education, Culture and Science, the Ministry for Health, Welfare and Sports, the European Commission (DG XII), and the Municipality of Rotterdam. The authors are grateful to the study participants, the staff from the Rotterdam Study and the participating general practitioners and pharmacists. The generation and management of GWAS genotype data for the Rotterdam Study (RS-I, RS-II, RS-III) was executed by the Human Genotyping Facility of the Genetic Laboratory of the Department of Internal Medicine, Erasmus MC, Rotterdam, The Netherlands. The GWAS datasets are supported by the Netherlands Organization of Scientific Research NWO Investments (Project number 175.010.2005.011, 911-03-012), the Genetic Laboratory of the Department of Internal Medicine, Erasmus MC, the Research Institute for Diseases in the Elderly (014-93-015; RIDE2), the Netherlands Genomics Initiative (NGI)/Netherlands Organization for Scientific Research (NWO) Netherlands Consortium for Healthy Aging (NCHA), project number 050-060-810. We thank Pascal Arp, Mila Jhamai,
Marijn Verkerk, Lizbeth Herrera and Marjolein Peters, MSc, and Carolina Medina-Gomez, MSc, for their help in creating the GWAS database, and Karol Estrada, PhD, Yurii Aulchenko, PhD, and Carolina Medina- Gomez, MSc, for the creation and analysis of imputed data.

© 2023 European Alzheimer’s & Dementia Biobank Mendelian Randomization (EADB-MR) Collaboration et al. *JAMA Network Open.*

QTLs/TWAS analyses. The results from EADB are in whole or in part based on data obtained from the AD Knowledge Portal (https://adknowledgeportal.synapse.org/). For MayoRNAseq, the study data were provided by the following sources: The Mayo Clinic Alzheimers Disease Genetic Studies, led by Dr. Nilufer Ertekin-Taner and Dr. Steven G. Younkin, Mayo Clinic, Jacksonville, FL using samples from the Mayo Clinic Study of Aging, the Mayo Clinic Alzheimers Disease Research Center, and the Mayo Clinic Brain Bank. Data collection was supported through funding by NIA grants P50 AG016574, R01 AG032990, U01 AG046139, R01 AG018023, U01 AG006576, U01 AG006786, R01 AG025711, R01 AG017216, R01 AG003949, NINDS grant R01 NS080820, CurePSP Foundation, and support from Mayo Foundation. Study data includes samples collected through the Sun Health Research Institute Brain and Body Donation Program of Sun City, Arizona. The Brain and Body Donation Program is supported by the National Institute of Neurological Disorders and Stroke (U24 NS072026 National Brain and Tissue Resource for Parkinsons Disease and Related Disorders), the National Institute on Aging (P30 AG19610 Arizona Alzheimers Disease Core Center), the Arizona Department of Health Services (contract 211002, Arizona Alzheimers Research Center), the Arizona Biomedical Research Commission (contracts 4001, 0011, 05-901 and 1001 to the Arizona Parkinson's Disease Consortium) and the Michael J. Fox Foundation for Parkinsons Research. For ROSMAP, the study data were provided by the Rush Alzheimer’s Disease Center, Rush University Medical Center, Chicago. Data collection was supported through funding by NIA grants P30AG10161 (ROS), R01AG15819 (ROSMAP; genomics and RNAseq), R01AG17917 (MAP), R01AG30146, R01AG36042 (5hC methylation, ATACseq), RC2AG036547 (H3K9Ac), R01AG36836 (RNAseq), R01AG48015 (monocyte RNAseq) RF1AG57473 (single nucleus RNAseq), U01AG32984 (genomic and whole exome sequencing), U01AG46152 (ROSMAP AMP-AD, targeted proteomics), U01AG46161(TMT proteomics), U01AG61356 (whole genome sequencing, targeted proteomics, ROSMAP AMP-AD), the Illinois Department of Public Health (ROSMAP), and the Translational Genomics Research Institute (genomic). Additional phenotypic data can be requested at www.radc.rush.edu. For MSBB, the data were generated from postmortem brain tissue collected through the Mount Sinai VA Medical Center Brain Bank and were provided by Dr. Eric Schadt from Mount Sinai School of Medicine. This work was supported by grants from the US National Institutes of Health (NIH NIA R21-G063130, NIA R01-AG054005, NIA R56-AG055824, and NIA U01-AG068880).

© 2023 European Alzheimer’s & Dementia Biobank Mendelian Randomization (EADB-MR) Collaboration et al. *JAMA Network Open.*

eReferences

1. Pirinen M. https://www.mv.helsinki.fi/home/mjxpirin/GWAS_course/material/GWAS3.html.
2. Morrison J, Knoblauch N, Marcus JH, Stephens M, He X. Mendelian randomization accounting for correlated and uncorrelated pleiotropic effects using genome-wide summary statistics. *Nat Genet*. Jul 2020;52(7):740-747. doi:10.1038/s41588-020-0631-4
3. Mounier N, Kutalik Z. Bias correction for inverse variance weighting Mendelian randomization. *bioRxiv*. 2021:2021.03.26.437168. doi:10.1101/2021.03.26.437168
4. Lauer MS, Anderson KM, Levy D. Influence of contemporary versus 30-year blood pressure levels on left ventricular mass and geometry: the Framingham Heart Study. *J Am Coll Cardiol*. Nov 1 1991;18(5):1287-94. doi:10.1016/0735-1097(91)90549-o
5. Lorell BH, Apstein CS, Weinberg EO, Cunningham MJ. Diastolic function in left ventricular hypertrophy: clinical and experimental relationships. *Eur Heart J*. Nov 1990;11 Suppl G:54-64. doi:10.1093/eurheartj/11.suppl_g.54
6. Scuteri A, Coluccia R, Castello L, Nevola E, Brancati AM, Volpe M. Left ventricular mass increase is associated with cognitive decline and dementia in the elderly independently of blood pressure. *Eur Heart J*. Jun 2009;30(12):1525-9. doi:10.1093/eurheartj/ehp133
7. Kuller LH, Margolis KL, Gaussoin SA, et al. Relationship of hypertension, blood pressure, and blood pressure control with white matter abnormalities in the Women's Health Initiative Memory Study (WHIMS)-MRI trial. *J Clin Hypertens (Greenwich)*. Mar 2010;12(3):203-12. doi:10.1111/j.1751-7176.2009.00234.x
8. Carnevale D, Mascio G, D'Andrea I, et al. Hypertension induces brain β-amyloid accumulation, cognitive impairment, and memory deterioration through activation of receptor for advanced glycation end products in brain vasculature. *Hypertension*. Jul 2012;60(1):188-97. doi:10.1161/hypertensionaha.112.195511
9. Hu H, Meng L, Bi YL, et al. Tau pathologies mediate the association of blood pressure with cognitive impairment in adults without dementia: The CABLE study. *Alzheimers Dement*. Jan 2022;18(1):53-64. doi:10.1002/alz.12377
10. Østergaard SD, Mukherjee S, Sharp SJ, et al. Associations between Potentially Modifiable Risk Factors and Alzheimer Disease: A Mendelian Randomization Study. *PLoS Med*. Jun 2015;12(6):e1001841; discussion e1001841. doi:10.1371/journal.pmed.1001841
11. Larsson SC, Traylor M, Malik R, Dichgans M, Burgess S, Markus HS. Modifiable pathways in Alzheimer's disease: Mendelian randomisation analysis. *Bmj*. Dec 6 2017;359:j5375. doi:10.1136/bmj.j5375
12. Andrews SJ, Fulton-Howard B, O'Reilly P, Marcora E, Goate AM. Causal Associations Between Modifiable Risk Factors and the Alzheimer's Phenome. *Ann Neurol*. Jan 2021;89(1):54-65. doi:10.1002/ana.25918
13. Zhang Q, Xu F, Wang L, Zhang WD, Sun CQ, Deng HW. Detecting potential causal relationship between multiple risk factors and Alzheimer's disease using multivariable Mendelian randomization. *Aging (Albany NY)*. Nov 7 2020;12(21):21747-21757. doi:10.18632/aging.103983
14. Lord J, Jermy B, Green R, et al. Mendelian randomization identifies blood metabolites previously linked to midlife cognition as causal candidates in Alzheimer's disease. *Proc Natl Acad Sci U S A*. Apr 20 2021;118(16)doi:10.1073/pnas.2009808118
15. Huang SY, Yang YX, Zhang YR, et al. Investigating Causal Relations Between Circulating Metabolites and Alzheimer's Disease: A Mendelian Randomization Study. *J Alzheimers Dis*. Mar 11 2022;doi:10.3233/jad-220050
16. Benn M, Nordestgaard BG, Frikke-Schmidt R, Tybjærg-Hansen A. Low LDL cholesterol, PCSK9 and HMGCR genetic variation, and risk of Alzheimer's disease and Parkinson's disease: Mendelian randomisation study. *Bmj*. Apr 24 2017;357:j1648. doi:10.1136/bmj.j1648

© 2023 European Alzheimer’s & Dementia Biobank Mendelian Randomization (EADB-MR) Collaboration et al. *JAMA Network Open.*

17. Williams DM, Finan C, Schmidt AF, Burgess S, Hingorani AD. Lipid lowering and Alzheimer disease risk: A mendelian randomization study. *Ann Neurol*. Jan 2020;87(1):30-39. doi:10.1002/ana.25642
18. Nordestgaard LT, Tybjærg-Hansen A, Nordestgaard BG, Frikke-Schmidt R. Body Mass Index and Risk of Alzheimer's Disease: A Mendelian Randomization Study of 399,536 Individuals. *J Clin Endocrinol Metab*. Jul 1 2017;102(7):2310-2320. doi:10.1210/jc.2017-00195
19. Zhou Y, Sun X, Zhou M. Body Shape and Alzheimer's Disease: A Mendelian Randomization Analysis. *Front Neurosci*. 2019;13:1084. doi:10.3389/fnins.2019.01084
20. Li X, Tian Y, Yang YX, et al. Life Course Adiposity and Alzheimer's Disease: A Mendelian Randomization Study. *J Alzheimers Dis*. 2021;82(2):503-512. doi:10.3233/jad-210345
21. Peloso GM, van der Lee SJ, Destefano AL, Seshardi S. Genetically elevated high-density lipoprotein cholesterol through the cholesteryl ester transfer protein gene does not associate with risk of Alzheimer's disease. *Alzheimers Dement (Amst)*. 2018;10:595-598. doi:10.1016/j.dadm.2018.08.008
22. Nordestgaard LT, Christoffersen M, Lauridsen BK, et al. Long-term Benefits and Harms Associated With Genetic Cholesteryl Ester Transfer Protein Deficiency in the General Population. *JAMA Cardiol*. Jan 1 2022;7(1):55-64. doi:10.1001/jamacardio.2021.3728
23. Kjeldsen EW, Thomassen JQ, Juul Rasmussen I, Nordestgaard BG, Tybjærg-Hansen A, Frikke-Schmidt R. Plasma high-density lipoprotein cholesterol and risk of dementia: observational and genetic studies. *Cardiovasc Res*. Mar 25 2022;118(5):1330-1343. doi:10.1093/cvr/cvab164
24. Andrews SJ, Goate A, Anstey KJ. Association between alcohol consumption and Alzheimer's disease: A Mendelian randomization study. *Alzheimers Dement*. Feb 2020;16(2):345-353. doi:10.1016/j.jalz.2019.09.086
25. Sproviero W, Winchester L, Newby D, et al. High Blood Pressure and Risk of Dementia: A Two-Sample Mendelian Randomization Study in the UK Biobank. *Biol Psychiatry*. Apr 15 2021;89(8):817-824. doi:10.1016/j.biopsych.2020.12.015
26. Ou YN, Yang YX, Shen XN, et al. Genetically determined blood pressure, antihypertensive medications, and risk of Alzheimer's disease: a Mendelian randomization study. *Alzheimers Res Ther*. Feb 9 2021;13(1):41. doi:10.1186/s13195-021-00782-y
27. Thomassen JQ, Tolstrup JS, Benn M, Frikke-Schmidt R. Type-2 diabetes and risk of dementia: observational and Mendelian randomisation studies in 1 million individuals. *Epidemiol Psychiatr Sci*. Apr 24 2020;29:e118. doi:10.1017/s2045796020000347
28. Benjamini Y, Drai D, Elmer G, Kafkafi N, Golani I. Controlling the false discovery rate in behavior genetics research. *Behav Brain Res*. Nov 1 2001;125(1-2):279-84. doi:10.1016/s0166-4328(01)00297-2

© 2023 European Alzheimer's & Dementia Biobank Mendelian Randomization (EADB-MR) Collaboration et al. *JAMA Network Open.*
