# Supplementary material for: Genetic Associations Between Modifiable Risk Factors and Alzheimer Disease
Source: JAMA Netw Open. 2023 May 17;6(5):e2313734. doi: 10.1001/jamanetworkopen.2023.13734 (PMC10193187; doi:10.1001/jamanetworkopen.2023.13734)
Supplement: Supplement 3. — Data Sharing Statement [file jamanetwopen-e2313734-s003.pdf]

Data Sharing Statement

Luo. Genetic Associations Between Modifiable Risk Factors and Alzheimer Disease. *JAMA Netw Open*. Published May 17, 2023. doi:10.1001/jamanetworkopen.2023.13734

Data
Data available: Yes
Data types: Other (please specify)
Additional Information: Summary statistics for genetic variants of the whole EADB dataset
How to access data: https://www.ebi.ac.uk/gwas/
When available: With publication

Supporting Documents
Document types: None

Additional Information
Who can access the data: Researchers whose proposed use of the data has been approved.
Types of analyses: For specific purposes.
Mechanisms of data availability: After approval of a proposal.
